# LKB1 Regulates Mitochondria-Dependent Presynaptic Calcium Clearance and Neurotransmitter Release Properties at Excitatory Synapses along Cortical Axons

**DOI:** 10.1371/journal.pbio.1002516

**Published:** 2016-07-18

**Authors:** Seok-Kyu Kwon, Richard Sando, Tommy L. Lewis, Yusuke Hirabayashi, Anton Maximov, Franck Polleux

**Affiliations:** 1 Columbia University Medical Center, Department of Neuroscience, Mortimer B. Zuckerman Mind Brain Behavior Institute, Kavli Institute for Brain Science, New York, New York, United States of America; 2 The Scripps Research Institute, Dorris Neuroscience Center, La Jolla, California, United States of America; University of Basel, SWITZERLAND

## Abstract

Individual synapses vary significantly in their neurotransmitter release properties, which underlie complex information processing in neural circuits. Presynaptic Ca^2+^ homeostasis plays a critical role in specifying neurotransmitter release properties, but the mechanisms regulating synapse-specific Ca^2+^ homeostasis in the mammalian brain are still poorly understood. Using electrophysiology and genetically encoded Ca^2+^ sensors targeted to the mitochondrial matrix or to presynaptic boutons of cortical pyramidal neurons, we demonstrate that the presence or absence of mitochondria at presynaptic boutons dictates neurotransmitter release properties through Mitochondrial Calcium Uniporter (MCU)-dependent Ca^2+^ clearance. We demonstrate that the serine/threonine kinase LKB1 regulates MCU expression, mitochondria-dependent Ca^2+^ clearance, and thereby, presynaptic release properties. Re-establishment of MCU-dependent mitochondrial Ca^2+^ uptake at glutamatergic synapses rescues the altered neurotransmitter release properties characterizing LKB1-null cortical axons. Our results provide novel insights into the cellular and molecular mechanisms whereby mitochondria control neurotransmitter release properties in a bouton-specific way through presynaptic Ca^2+^ clearance.

## Abstract 2

The capture of mitochondria at individual presynaptic boutons plays a synapse-specific role in regulating neurotransmission through the ability to regulate presynaptic calcium levels.

## Author Summary

Mitochondria perform many important biological functions, ranging from ATP production through oxidative phosphorylation to calcium clearance and lipid biosynthesis. In neurons of the mammalian cerebral cortex, we discovered that only ~50% of presynaptic boutons (the site where neurotransmission occurs) are associated with mitochondria while ~50% are not. Our results demonstrate that the presence of a presynaptic mitochondria plays a key role in calcium homeostasis and, thereby, regulates the properties of neurotransmitter release. We identify a protein kinase called LKB1, which regulates the ability of presynaptic mitochondria to clear calcium, by controlling the abundance of the mitochondrial calcium uniporter—a Ca^2+^-selective ion channel located in the mitochondria’s inner membrane. Our results suggest that mitochondrial capture at individual presynaptic boutons along cortical axon plays a synapse-specific role in regulating neurotransmission through the ability to regulate presynaptic calcium homeostasis.

## Introduction

Neurotransmitter release properties vary greatly between presynaptic terminals of different neurons, but also between presynaptic release sites of the same neuron. At nerve terminals, rapid calcium (Ca^2+^) influx through voltage-gated Ca^2+^ channels (VGCC) triggers exocytosis of neurotransmitter vesicles on a sub-millisecond timescale. Over the past two decades, significant progress has been made in understanding how calcium sensors, synaptotagmins, drive vesicle exocytosis by binding to phospholipids and SNARE machinery [1–4]. Interestingly, several studies revealed that action potential (AP)-evoked presynaptic Ca^2+^ signals can also vary drastically between different boutons along the same axons [5–8]. For example, in cortical pyramidal neurons, individual presynaptic release sites distributed along a single axon have different patterns of Ca^2+^ dynamics and neurotransmitter release probability depending on the postsynaptic target cells [5, 9–13]. However, the cellular and molecular pathways regulating Ca^2+^ dynamics in a synapse-specific way are poorly understood.

In various cell types, mitochondria perform critical biological functions, including ATP production through oxidative phosphorylation, Ca^2+^ clearance, and lipid biogenesis [14,15]. These pathways have been intensely studied in non-neuronal cells and also in the context of neurodegeneration [16], but the roles of mitochondria during neuronal development and physiological synaptic function in adult axons are still poorly understood. It has been suggested that mitochondria are involved in presynaptic Ca^2+^ clearance, but the impact on modulation of neurotransmitter release varies in different species and neuron subtypes [17–24]. Mitochondria can also regulate presynaptic release properties through their metabolic functions [25]. Nevertheless, the signaling pathways regulating presynaptic mitochondrial function in this context are largely unknown.

We and others identified that the serine/threonine kinase LKB1 (Liver Kinase B1 also called STK11 or Par4) is a master regulator of axon morphogenesis in the mammalian central nervous system (CNS). LKB1 is necessary and sufficient for axon formation in long-range projecting, cortical pyramidal neurons [26,27]. More recently, we also found that at later stages of axon development, LKB1 plays an essential role in terminal axon branching in vivo by regulating presynaptic mitochondria capture at nascent boutons [28]. These results raised a central unresolved question regarding the relevance of presynaptic mitochondria in axon morphogenesis.

Here, we report that the presence or absence of presynaptic mitochondria represent a key component of presynaptic Ca^2+^ homeostasis and neurotransmitter release properties in a synapse-specific manner. In addition, we identified that the LKB1 kinase controls presynaptic Ca^2+^ homeostasis through regulation of the abundance of the mitochondrial calcium uniporter (MCU). Disruption of this signaling pathway leads to increased presynaptic Ca^2+^ accumulation and drastic changes in neurotransmitter release properties, including (1) increased rate of spontaneous vesicle fusion, (2) augmentation of asynchronous mode of evoked neurotransmitter release, (3) abrogation of short-term synaptic depression during trains of action potentials (APs), and (4) an increase in the frequency of action potential burst firing. Our results identify a new LKB1-dependent signaling pathway regulating neurotransmitter release properties in neurons through the control of mitochondria-dependent presynaptic Ca^2+^ clearance.

## Results

### Presence or Absence of Mitochondria at Presynaptic Boutons Correlates with Cytosolic Ca^2+^ Dynamics during Repetitive Stimulation of Neurotransmitter Release

**Fig 1:**
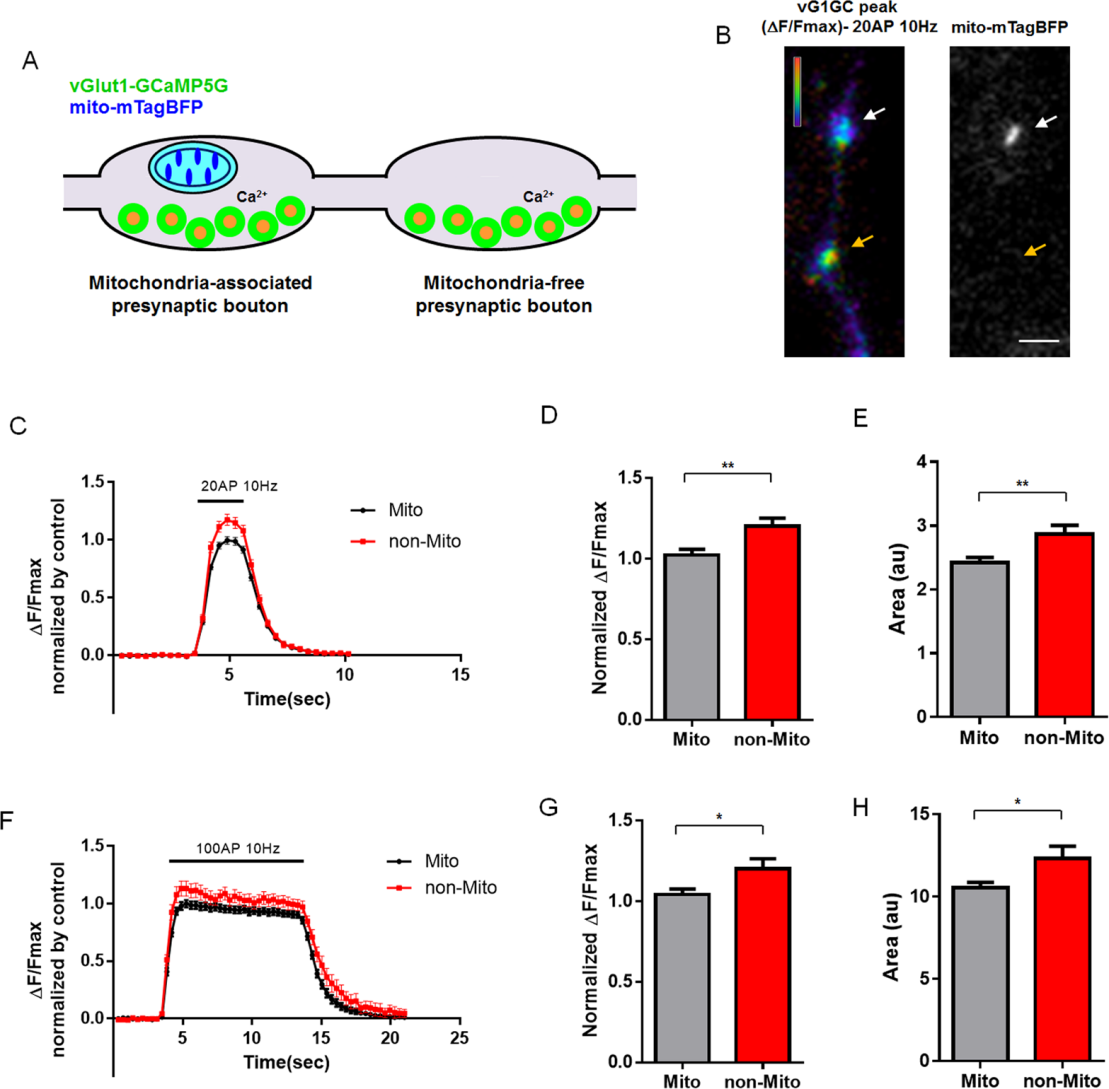
Mitochondria-free presynaptic boutons have increased cytoplasmic Ca^2+^ during repetitive stimulation. (A) Mitochondria-occupied and -free presynaptic Ca^2+^ dynamics were monitored using vGlut1-GCaMP5G and mito-mTagBFP in axons of cultured cortical neurons following ex utero electroporation at E15.5 and imaged at 15–17 DIV. (B) Representative image of vGlut1-GCaMP5G peak signal during 20 AP at 10 Hz and mito-mTagBFP. vGlut1-GCaMP5G image is converted to ratio view normalized by Fmax signal followed by ionomycin (5 μM) incubation. White arrows point to mitochondria-associated presynaptic site and orange arrows point to mitochondria-free presynaptic site. (C–H) vGlut1-GCaMP5G signals in mitochondria-free boutons show significantly increased normalized peak values and total charge transfer (area under curve) during repetitive stimulation (20 AP and 100 AP at 10 Hz). *n* = 62 for mito-associated boutons, and 40 for mito-free boutons from 14 neurons in 20 AP condition. *n* = 34 for mitochondria-associated boutons and 23 for mitochondria-free boutons from 9 neurons in 100 AP condition. *p < 0.05 and ** *p* < 0.01, Mann-Whitney test. Scale bar = 3 μm. Individual values are available in S1 Data.

Mitochondria are associated with about half of presynaptic sites in axons of mature pyramidal cortical neurons (**S3D Fig**) [21,29]. In order to study the function of mitochondria in Ca^2+^ homeostasis at individual presynaptic boutons, we used a genetically encoded calcium sensor, GCaMP5G [30], fused with vesicular glutamate transporter 1 (vGlut1; SLC17A7), a transmembrane protein enriched at the membrane of presynaptic vesicles in cortical pyramidal neurons (**S1A and S1C Fig**). This vGlut1-GCaMP5G fusion protein has improved sensitivity for monitoring presynaptic cytoplasmic Ca^2+^ levels compared to non-targeted, cytosolic GCaMP5G (**S2 Fig**). This plasmid was introduced with mitochondrial-targeted blue fluorescent protein mTagBFP (mito-mTagBFP) and vGlut1-mCherry via ex utero cortical electroporation (EUCE) at E15.5. Following dissociation and plating, cortical neurons were imaged at 15–18 d in vitro (DIV) under evoked APs using a concentric bipolar electrode. This system allowed us to measure presynaptic, cytoplasmic Ca^2+^ ([Ca^2+^]_c_) dynamics at individual presynaptic boutons associated or not with mitochondria along the same axon segments (**Fig 1A**). Interestingly, using this approach, we found that during moderate trains of AP stimulation (20 AP at 10 Hz), the peak intensity of vGlut1-GCaMP5G signals and the total charge transfer (area under curve) were significantly increased at presynaptic sites not associated with mitochondria (**Fig 1B–1E**) compared to presynaptic boutons associated with mitochondria along the same axon segments. Similar results were observed following 100 AP stimulation (**Fig 1F–1H**), as well as a whole range of stimulation conditions (10 AP, 20 AP, 50 AP; **S3 Fig**). These data suggest that differential presynaptic Ca^2+^ dynamics characterizing individual synapses along the same axon could be influenced by the presence or absence of presynaptic mitochondria. Also, these results suggest that Ca^2+^-dependent presynaptic release properties such as asynchronous release and short-term synaptic plasticity could be regulated by the presence or absence of mitochondria at specific presynaptic boutons along cortical axons.

### Mitochondria-Free Presynaptic Boutons Show Increased Neurotransmitter Release Compared to Mitochondria-Associated Boutons

**Fig 2:**
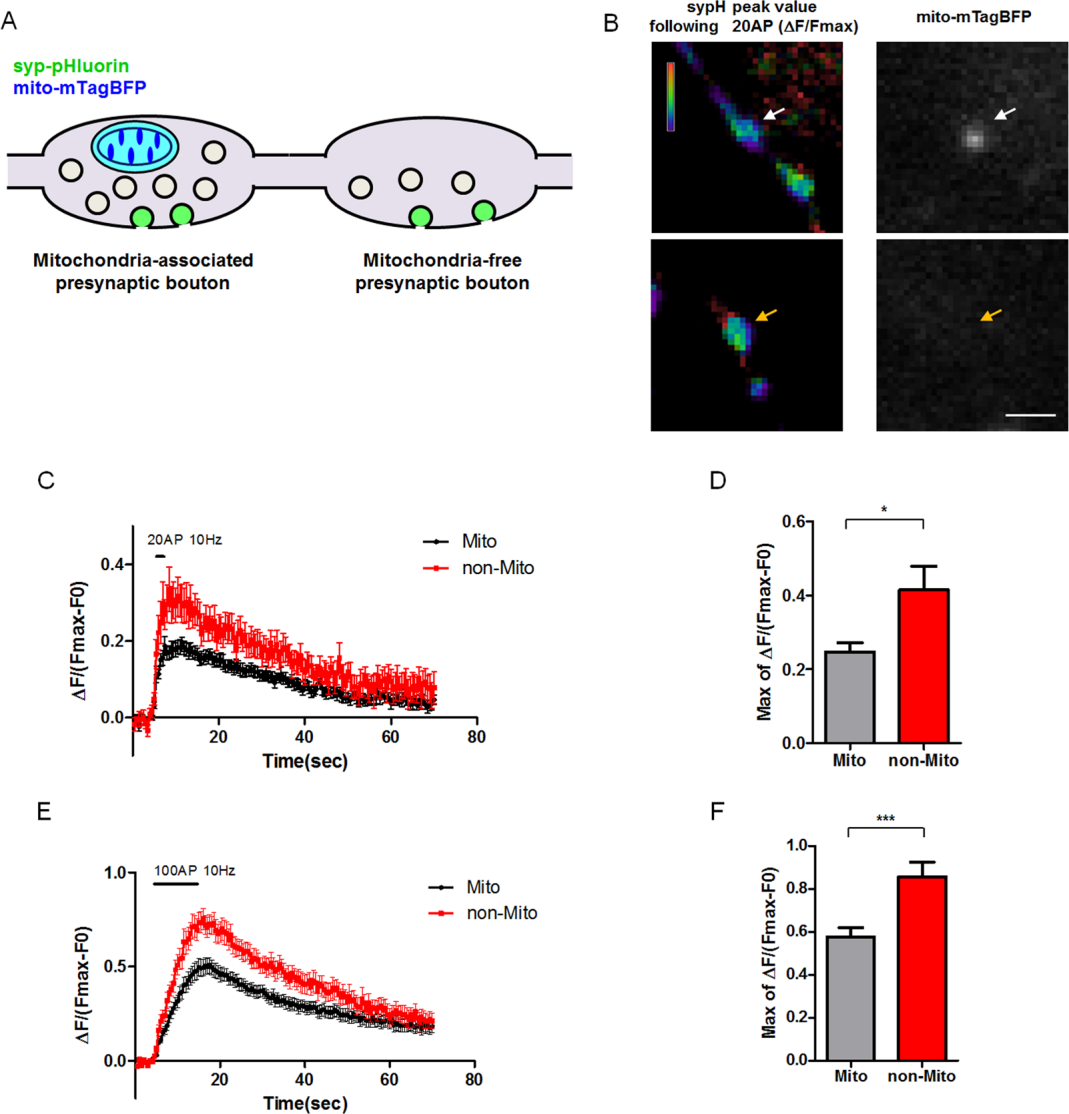
Mitochondria-free presynaptic boutons have increased neurotransmitter release during repetitive stimulation. **(A)** Presynaptic release properties at mitochondria-associated and -free boutons were monitored using synaptophysin-pHluorin and mito-mTagBFP in cultured neurons at 15–17 DIV. **(B)** Representative image of sypH peak value following 20 AP at 10 Hz. sypH images were visualized by ratio of the peak image normalized by Fmax signal. Fmax of sypH was obtained by NH_4_Cl (50mM) incubation. White arrows indicate mitochondria-associated presynaptic site and orange arrows indicate mitochondria-free presynaptic site. **(C and E)** Graphs of exocytic response at presynaptic sites with or without mitochondria show increased release in mitochondria-free bouton following both 20 and 100 AP stimuli. **(D and F)** Quantification of normalized peaks from C and E. Presynaptic sites without mitochondria have significantly increased release. *n* = 26 for mitochondria-associated boutons and 15 for mitochondria-free boutons from 15 neurons in 20AP condition. *n* = 31 for mitochondria-associated boutons and 17 for mitochondria-free boutons from 15 neurons in 100AP condition. * *p* < 0.05, Mann-Whitney test. Scale bar = 10μm. Individual values are available in **S1 Data**.

Presynaptic Ca^2+^ triggers neurotransmitter release through Ca^2+^-dependent vesicle exocytosis. Therefore, one prediction of the elevated presynaptic Ca^2+^ accumulation at mitochondria-free boutons is that it could lead to increased neurotransmitter release properties. In order to monitor synaptic vesicle release at individual presynaptic boutons, we employed a recently developed fusion protein made with the pHluorin tag fused to luminal domain of Synaptophysin (syp-pHluorin [31]). pHluorin is a pH-sensitive green fluorescent protein (GFP) whose fluorescence is quenched inside the lumen of a synaptic vesicle due to the acidic pH, and fluorescence is induced when it is exposed to the extracellular pH following exocytosis [32–35]. This probe allowed us to measure neurotransmitter release at individual presynaptic boutons along axons of layer 2/3 cortical neurons. We expressed syp-pHluorin, synaptophysin-mCherry, and mito-mTagBFP in cortical neurons by ex utero electroporation, and at 15-18DIV, and imaged syp-pHluorin signals using the same stimulation protocol as described above (20 AP or 100 AP at 10 Hz). Then, the maximum syp-pHluorin signal was measured for normalization following application of 50 mM NH_4_Cl application (**Fig 2A and 2B**). This normalization is required because individual presynaptic responses vary greatly along the axons, and de-acidification by NH_4_Cl normalizes not only for potential difference in syp-pHluorin expression levels but also total pool size of individual boutons ([36]; and see **S4 Fig**). Therefore, using this standard normalization approach, one can reliably estimate the amount of neurotransmitter vesicle exocytosis as a fraction of the total pool size at individual boutons (**S4 Fig**). Strikingly, mitochondria-free presynaptic boutons showed significantly increased synaptic vesicle fusion compared to presynaptic boutons associated with mitochondria following both types of repetitive stimulation (both 20 AP or 100 AP at 10 Hz; **Fig 2C–2F**). The peak of normalized syp-pHluorin intensity was 50%–80% higher at mitochondria-free presynaptic boutons compared to presynaptic boutons associated with mitochondria. These results are in agreement with the higher cytoplasmic Ca^2+^ levels measured with vGlut1-GCaMP5G (see **Fig 1**) observed at mitochondria-free presynaptic boutons compared to mitochondria-associated presynaptic boutons. Overall, these data suggest a model whereby the presence of mitochondria is associated with significant differences in neurotransmitter release properties in a bouton-specific manner possibly through mitochondria-dependent Ca^2+^ clearance.

### MCU-Dependent Ca^2+^ Import Is Required for Presynaptic Ca^2+^ Clearance, and LKB1 Loss-of-Function Leads MCU Reduction

**Fig 3:**
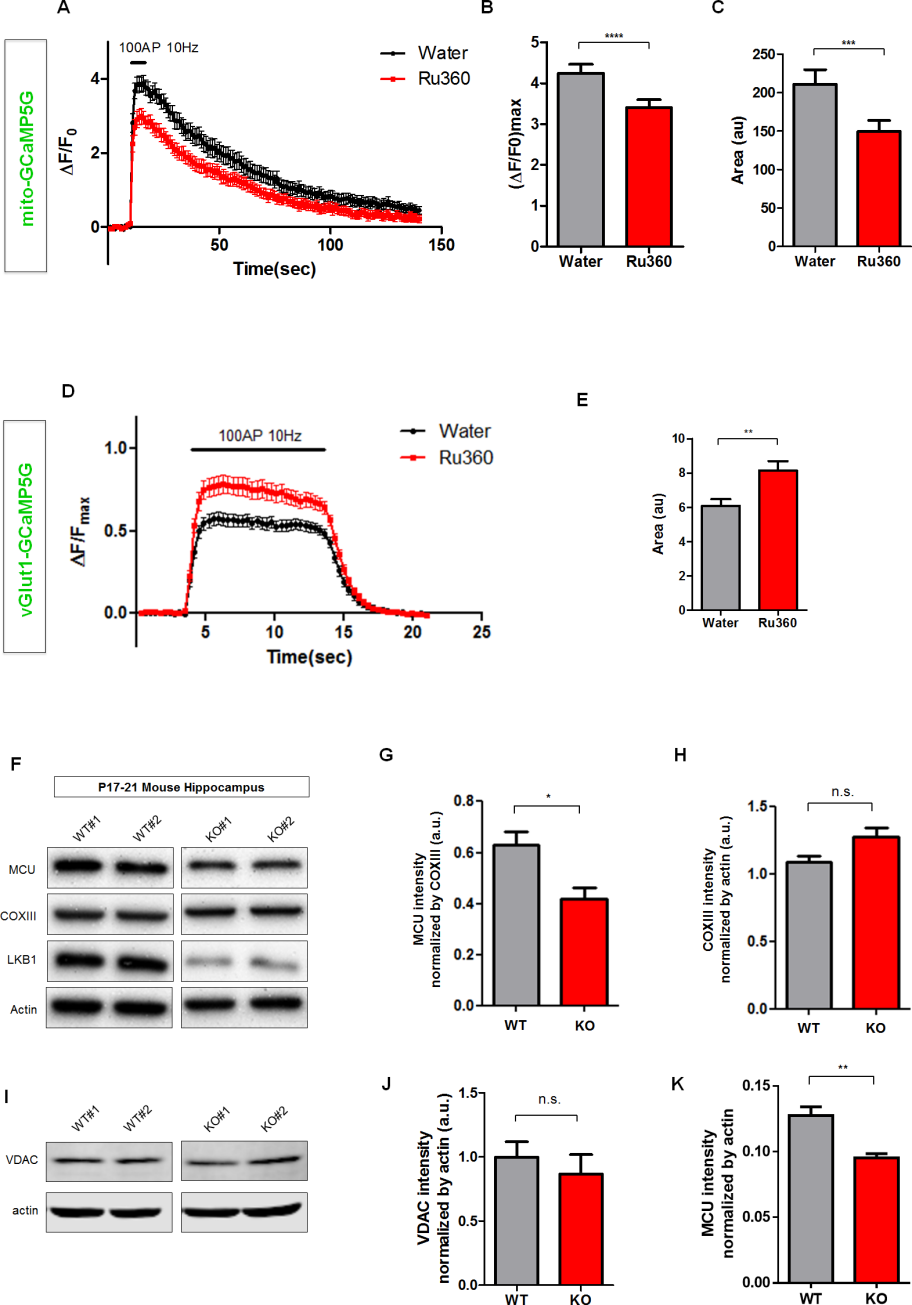
MCU is required for normal presynaptic Ca^2+^ clearance and MCU expression is decreased in LKB1-null hippocampi. **(A–C)** MCU inhibitor Ru360 reduced mitochondrial calcium influx during repetitive stimulation (100 AP at 10 Hz). Mito-GCaMP5G signals at the same presynaptic boutons were imaged before and after Ru360 (10 μM) incubation. Peak value and total charge transfer (area under curve) were significantly reduced by Ru360 application. *n* = 40 for water, 40 for Ru360 from 11 neurons. **** *p* < 0.0001, *** *p* < 0.001, Paired *t* test. **(D and E)** Acute inhibition of MCU by application of MCU inhibitor Ru360 (10 μM applied 3 min before and during imaging) increased presynaptic calcium (vGlut1-GCaMP5G) accumulation during repetitive stimulation with 100 AP at 10 Hz. *n* = 22 for water, 21 for Ru360 from 6 neurons. ** *p* < 0.01, Mann-Whitney test. **(F–K)** MCU expression level was significantly reduced in LKB1 knockout (Nex-Cre;LKB1^F/F^) whole-cell lysate from hippocampus, whereas other mitochondrial components, cytochrome c oxidase III (COXIII) and voltage-dependent anion channel (VDAC), were not significantly changed. **(G)** MCU intensity normalized by COXIII. **(H)** COXIII intensity normalized by actin. (**I**) Western blot for VDAC and Actin. **(J)** VDAC intensity normalized by actin. *n* = 5 for wild type (WT), 5 for knockout (KO). (**K**) Quantification of MCU expression in WT and LKB1 KO normalized to actin. * *p* < 0.05, Mann-Whitney test. Individual values are available in **S1 Data**.

Mitochondria uptake of cytoplasmic calcium is mediated through the mitochondrial calcium uniporter (MCU; also called ccdc109a), an ion channel selective for Ca^2+^ and present in the inner mitochondrial membrane [37–39]. In order to test more directly if the presence of mitochondria at specific presynaptic bouton and in particular if MCU-dependent mitochondrial Ca^2+^ import is required acutely for proper presynaptic Ca^2+^ homeostasis in cortical axons, we first took a pharmacological approach. We tested if acute inhibition of MCU function leads to increased [Ca^2+^]_c_ accumulation during synaptic transmission. To do this, we examined the effects of Ru360, the membrane permeable MCU inhibitor [40], on mitochondrial [Ca^2+^]_m_ and presynaptic [Ca^2+^]_c_ dynamics. In order to monitor Ca^2+^ dynamics inside the mitochondrial matrix ([Ca^2+^]_m_), we fused GCaMP5G to a short mitochondrial targeting peptide isolated from cytochrome c subunit VIII (mito-GCaMP5G) (**S1B and S1D Fig**). Together with vGlut1-GCaMP5G, we observed that acute application of Ru360 (10 μM, 3 min pre-incubation before imaging) significantly reduced mito-GCaMP5G signals and increased vGlut1-GCaMP5G signals during 100 AP stimulation (**Fig 3A–3E**), suggesting that MCU function is required for proper presynaptic [Ca^2+^]_c_ clearance under sustained forms of evoked neurotransmitter release.

The serine-threonine kinase LKB1 plays an important role in axon morphogenesis through regulation of presynaptic mitochondria capture [28]. Therefore, we tested if LKB1 also regulates mitochondrial function in more mature cortical neurons. First, we examined the expression level of MCU in the brain of LKB1-deficient mice. We used a pyramidal neuron-specific Nex^Cre^ mouse line [41] which induces recombination throughout the dorsal telencephalon including the hippocampus and neocortex during development in vivo [28]. In order to probe a region of the forebrain with reduced cell type diversity, we collected hippocampi from P17–21 Nex^Cre^;LKB1^F/F^ cKO. Subunit III of cytochrome c oxidase (COXIII), a component of the mitochondrial electron transport chain, and the voltage-dependent anion channel (VDAC) on the outer mitochondrial membrane did not show altered expression by quantitative western blot (**Fig 3F–3J**) and COXIII was therefore used to normalize MCU expression levels (**Fig 3G**). Interestingly, MCU expression level was significantly reduced in Nex^Cre^;LKB1^F/F^ cKO compared to control littermates (**Fig 3G and 3K**). These results suggest that LKB1 might regulate presynaptic Ca^2+^ homeostasis through mitochondrial Ca^2+^ import during synaptic transmission.

### MCU Reduction in LKB1-Null Axons Leads to Impaired Mitochondrial Ca^2+^ Import

We recently showed that LKB1 regulates axon branching by controlling presynaptic mitochondrial capture during early stages of axon morphogenesis in vitro (5–7 DIV) and in vivo (P7–P15). However, we found that at later stages of axon development i.e. after the peak of axon branching and synaptogenesis, mitochondria are being successfully captured at presynaptic sites in LKB1-null axons (**S5 Fig**). Along mature cortical axons (15–18 DIV), the same proportion of mitochondria are located at presynaptic boutons in both control and LKB1-deficient axons (39% +/- 2.9 in control; 44% +/- 4.5 in LKB1-null axons), and ~50% of vGlut1+ presynaptic boutons are occupied by mitochondria in layer 2/3 cortical neurons of both genotypes (**S5 Fig**) [42]. In order to compare the function of presynaptic mitochondria in the regulation of [Ca^2+^]_c_ homeostasis in control and LKB1-deficient cortical neurons, we restricted our analysis to presynaptic sites occupied by mitochondria.

We next tested if conditional deletion of LKB1 in postmitotic cortical neurons using Nex^Cre^ affected presynaptic development using an in vitro heterologous assay where presynaptic boutons are induced by Neuroligin-1 expression in COS7 cells [43] (**S6A and S6B Fig**) or in vivo using presynaptic reporter expression in layer 2/3 pyramidal neurons (**S6E–S6H Fig**). We found that LKB1-deficient axons form vGlut1-positive presynaptic boutons at the same density and similar size onto Neuroligin-1-expressing COS7 cells compared to control axons (**S6C and S6D Fig**). As previously reported [28], LKB1 is required for terminal axon branching thereby affecting the total number of presynaptic boutons made by axons of layer 2/3 pyramidal neurons in vivo (**S6E Fig**). However, LKB1 deletion does not affect the linear density of presynaptic boutons along the remaining axon branches (**S6F–S6H Fig**). We conclude that LKB1 is required for terminal axon branching but is not required for presynaptic development at early [28] or late stages of cortical axon maturation (**S6 Fig**).

**Fig 4:**
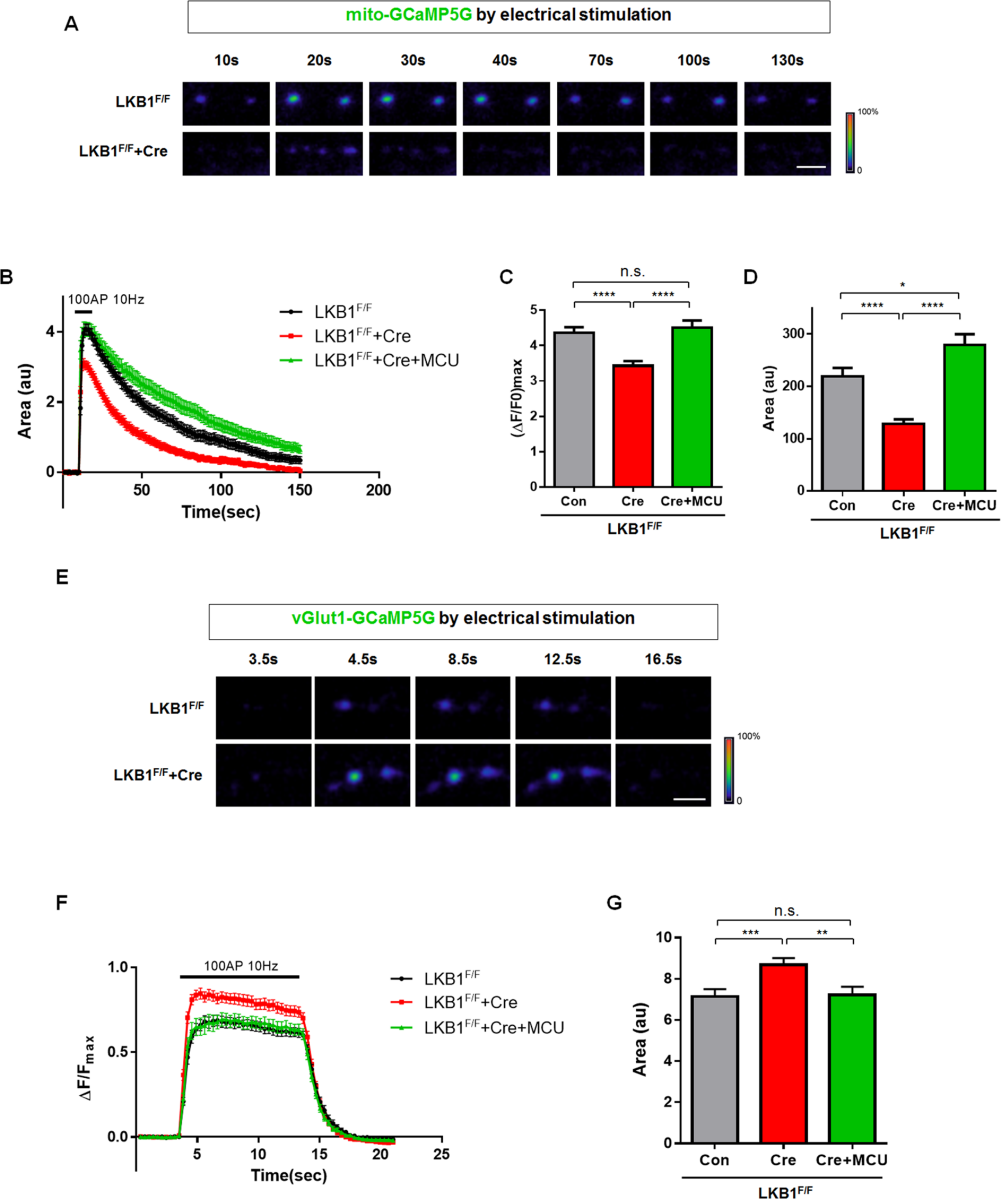
LKB1 regulates mitochondrial matrix Ca^2+^ import and MCU-dependent cytoplasmic Ca^2+^ clearance at presynaptic sites. **(A–D)** Loss of LKB1 reduced intra-mitochondrial calcium influx during evoked release imposed by 100 AP at 10 Hz and MCU overexpression rescued the defect. **(A)** Captured images from mito-GCaMP5G signals at vGlut1-mCherry presynaptic sites (not shown). **(B–D)** LKB1 deletion decreased maximum intensity and total charge transfer (area under the curve) of mito-GCaMP5G signals and MCU overexpression on LKB1-deficient neurons rescued intra-mitochondrial calcium import. *n* = 72 for control from 21 neurons, 75 for LKB1-null from 22 neurons, and 51 for LKB1-deletion+MCU from 12 neurons. **** *p* < 0.0001, * *p* < 0.05, Mann-Whitney test. Scale bar = 10 μm. **(E–G)** Conditional LKB1 deletion in cortical pyramidal neurons increased presynaptic Ca^2+^ during evoked release imposed by 100 AP at 10 Hz and MCU overexpression restored the defect. **(E)** Captured images from mitochondria-associated vGlut1-GCaMP5G signal at presynaptic boutons at specific times before and after stimulation. **(F–G)** LKB1-null neurons show significant increase of total intensity of vGlut1-GCaMP5G (area under the curve) and the increased intensity returned to control level following MCU overexpression. *n* = 38 for control from 11 neurons, 37 for LKB1-deletion from 10 neurons, 35 for LKB1-deletion+MCU from 12 neurons. * *p* < 0.05, *** *p* < 0.001. Mann-Whitney test. Individual values are available in **S1 Data**.

Then, we tested if the decreased MCU level observed in LKB1 mutants leads to defective mitochondrial calcium import ([Ca^2+^]_m_). To this end, three vectors encoding mito-GCaMP5G ([Ca^2+^]_m_), a presynapse-targeted mCherry (vGlut1-mCherry) and Cre recombinase were introduced into the cortex of LKB1^F/F^ mouse embryos [26,44] via ex utero cortical electroporation at E15.5. At 15–18 DIV we monitored intra-mitochondrial calcium dynamics ([Ca^2+^]_m_) by time-lapse microscopy when presynaptic release was induced using the stimulation protocol described above (100 AP at 10 Hz). Interestingly, LKB1-null axons showed a significant reduction in peak intensity of mito-GCaMP5G signal (**Fig 4A–4C**) and the total amount of Ca^2+^ imported inside the mitochondrial matrix (total [Ca^2+^]_m_ calculated as the area under the curve in **Fig 4D**) compared to control.

Next, we tested whether the decrease of MCU expression observed in LKB1-null neurons causes altered presynaptic [Ca^2+^]_c_ homeostasis. To this aim, we validated that a plasmid expressing MCU-HA is properly targeted to mitochondria in cortical axons (**S7 Fig**). We identified a low level of MCU expression (0.2 mg/ml) in LKB1-null cortical neurons that restored the peak intensity of [Ca^2+^]_m_ following 100 AP stimulation back to control levels (**Fig 4B and 4C**) and also caused significant increase of total [Ca^2+^]_m_ compared to control (**Fig 4D**).

### LKB1 Is Required for MCU-Dependent Presynaptic Ca^2+^ Homeostasis during Spontaneous and Evoked Release

We next tested if mitochondria-dependent presynaptic calcium dynamics is regulated by LKB1. Using co-expression of vGlut1-GCaMP5G ([Ca^2+^]_c_) and a mitochondria-targeted mCherry (mito-mCherry) allowed us to monitor presynaptic cytoplasmic Ca^2+^ ([Ca^2+^]_c_) dynamics during neurotransmission. We augmented excitatory synaptic network activity in these cortical cultures by applying a GABA_A_ receptor antagonist, picrotoxin. Interestingly, we found that LKB1-null axons show a significant increase in the maximal intensity of [Ca^2+^]_c_ during spontaneous bursts of presynaptic release compared to presynaptic sites in control axons (**S8 Fig**). Furthermore, [Ca^2+^]_c_ decay kinetics (t_1/2_) was more than two times slower in presynaptic sites of LKB1-null axons compared to control (**S8D Fig**). In these conditions, when cultured neurons are subject to spontaneous activity, the observed increased decay time of presynaptic [Ca^2+^]_c_ might be due to prolonged burst width observed during spontaneous network activity using electrophysiological recordings (**S11 Fig**). These results suggest a defect in presynaptic Ca^2+^ clearance in LKB1-null axons compared to control.

**Fig 5:**
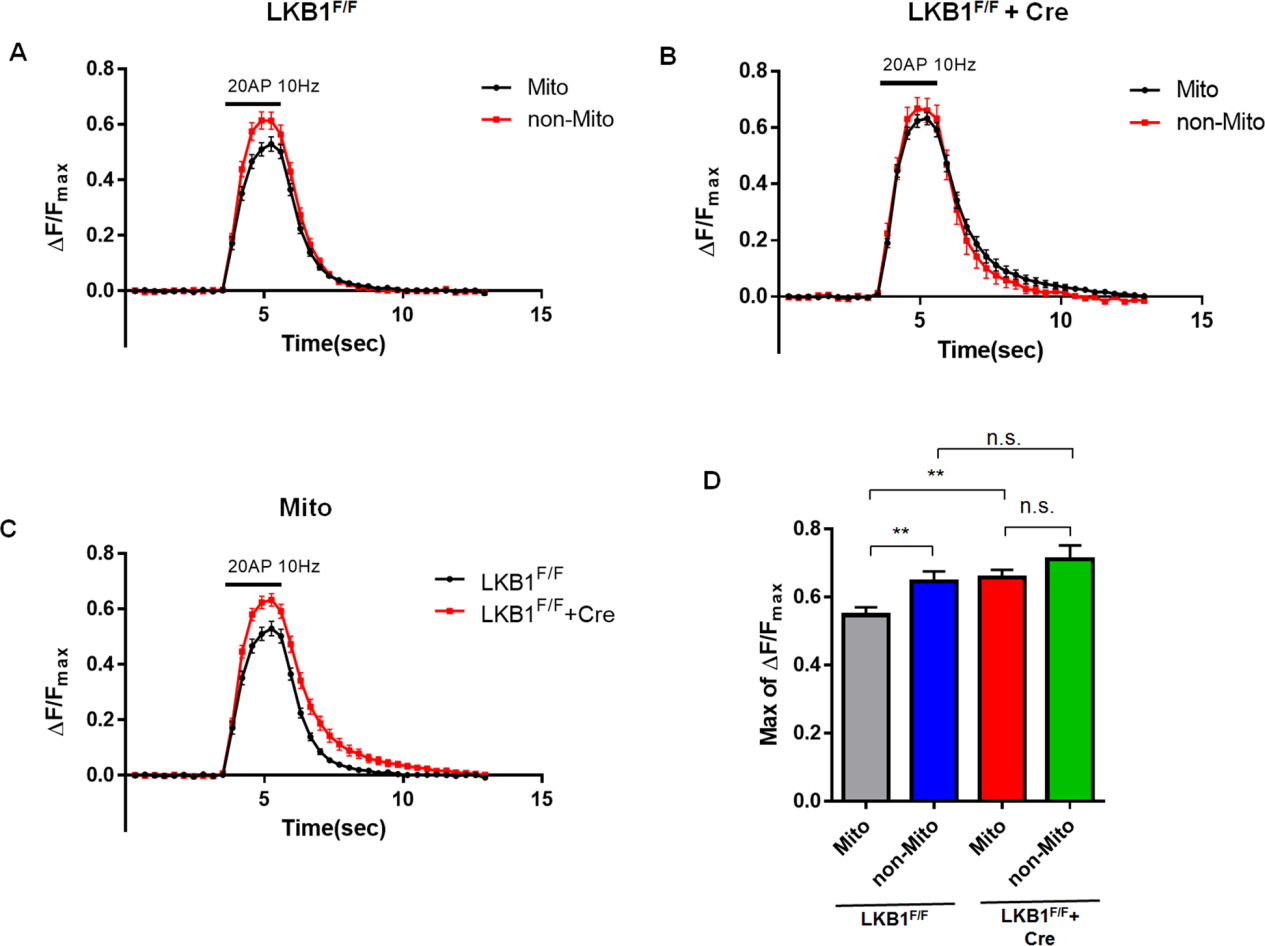
Defective presynaptic Ca^2+^ dynamics occurs only in mitochondria-associated boutons in LKB1-null neurons. **(A)** WT neurons display significantly increased vGlut1-GCaMP5G peak values in mitochondria-free boutons compared to mitochondria-associated boutons during repetitive stimulation (20 AP at 10 Hz). **(B)** LKB1-deficient neurons display no significant difference in presynaptic calcium accumulation (vGlut1-GCaMP5G) between mitochondria-associated and mitochondria–free boutons. **(C)** Following 20 AP at 10 Hz stimulation, mitochondria-associated presynaptic boutons in LKB1-null axons show significantly increased presynaptic Ca^2+^ (vGlut1-GCaMP5G) compared to WT boutons. **(D)** Histogram of peak values for each condition. *n* = 34 for mito-associated boutons, and 36 for mito-free boutons from 16 neurons in WT. *n* = 51 for mitochondria-associated boutons and 19 for mitochondria-free boutons from 18 neurons in LKB1-null. ** *p* < 0.01, Mann-Whitney test. Individual values are available in **S1 Data**.

Since picrotoxin-induced bursts of network activity are triggered by variable and unknown numbers of APs, we further examined presynaptic [Ca^2+^]_c_ dynamics during induction of repetitive APs using electrical stimulation. In this case, network responses were blocked with glutamate receptor antagonists, CNQX and APV, and presynaptic Ca^2+^ levels were measured during trains of 100APs as shown in **S1 Fig**. Again, under these conditions, presynaptic [Ca^2+^]_c_ elevated to significantly higher levels in LKB1-null axons compared to control axons (**Fig 4E–4G**). In addition, although wild-type LKB1 rescued the impaired presynaptic [Ca^2+^]_c_, but two catalytically inactive forms of LKB1 (LKB1^D194A^ and LKB1^K78A^) did not restore the phenotype (**S9 Fig**). These results demonstrate that LKB1 signaling is required for proper presynaptic [Ca^2+^]_c_ clearance. More interestingly, MCU overexpression rescued [Ca^2+^]_c_ elevation in LKB1-deficient cortical neurons back to control levels (**Fig 4E–4G**). In addition, LKB1-null neurons showed no difference between mitochondria-free and mitochondria-associated presynaptic [Ca^2+^]_c_, which are significantly higher than mitochondria-associated [Ca^2+^]_c_ of wild type (WT) neurons (**Fig 5**). These results suggest that, unlike in control WT neurons, in LKB1-null neurons, presynaptic [Ca^2+^]_c_ dynamics are not different between boutons associated with mitochondria or not. Therefore, we conclude that LKB1 regulate presynaptic [Ca^2+^]_c_ homeostasis through mitochondria-dependent calcium buffering and that MCU is a central effector for presynaptic [Ca^2+^]_c_ homeostasis downstream of LKB1 in axons of cortical pyramidal neurons.

### Aberrant Presynaptic Ca^2+^ Homeostasis in Axons of LKB1-Null Neurons Leads to Defective Neurotransmitter Release

Since presynaptic Ca^2+^ influx through VGCC plays a critical role in vesicle exocytosis and recycling [1–4], we reasoned that the aberrant presynaptic Ca^2+^ homeostasis observed in LKB1-null axons should lead to strong defects in neurotransmitter release. Based on published evidence, elevation of resting and residual presynaptic [Ca^2+^]_c_ levels should lead to (1) increased frequency of spontaneous miniature Excitatory Post-Synaptic Currents (mEPSCs), (2) augmentation of “slow” asynchronous release, and (3) altered facilitation/depression of release during high-frequency stimulation [12,45–49]. We tested all three predictions using electrophysiological approaches. Dissociated LKB1^F/F^ cortical neuron cultures were infected with ΔCre cDNA lacking the enzymatic domain of Cre recombinase or full-length Cre-encoding lentiviruses at 3–5 DIV. Spontaneous synaptic currents were monitored in whole-cell voltage-clamp mode at 15–18 DIV in dissociated cortical pyramidal neurons.

**Fig 6:**
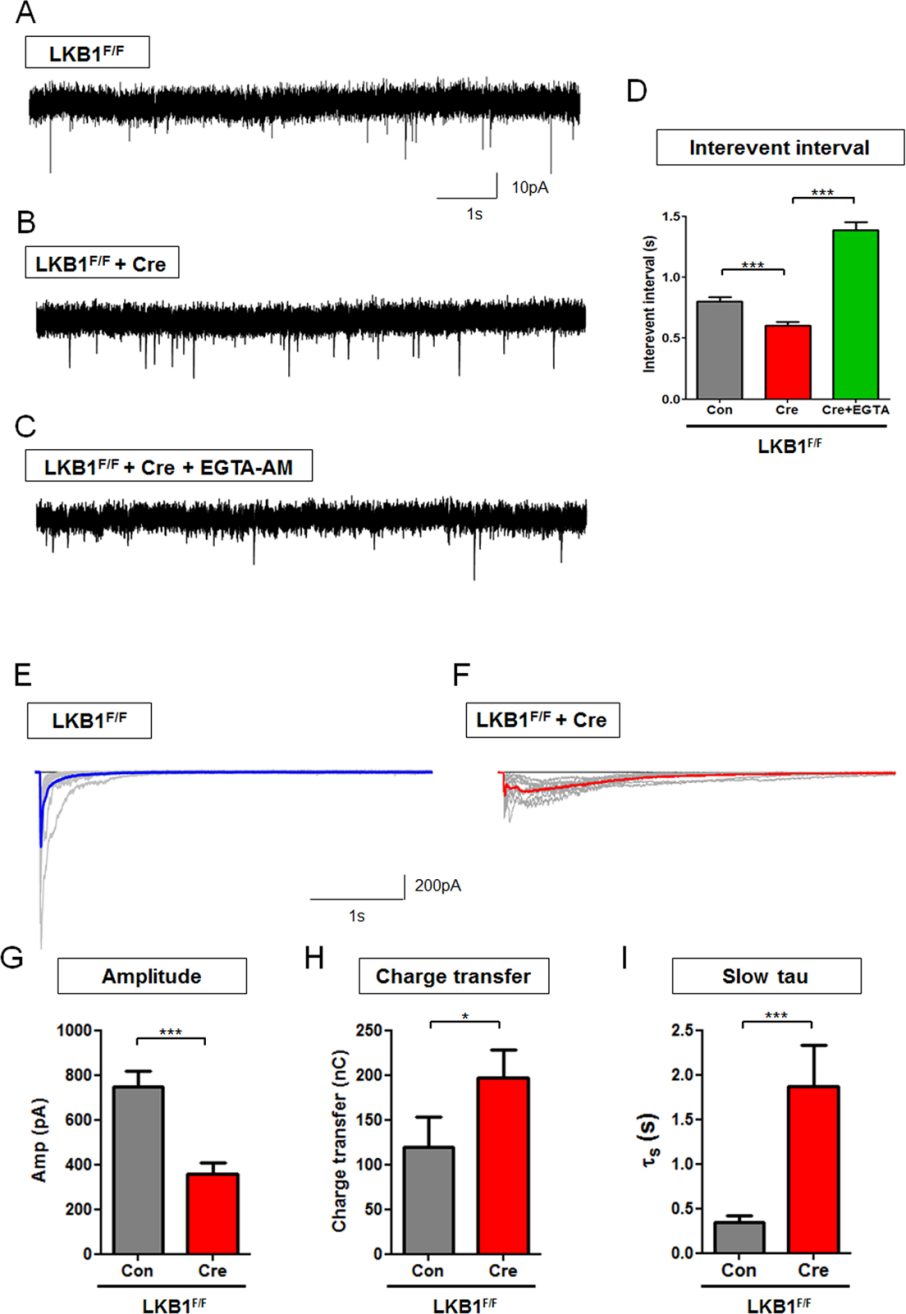
Conditional deletion of LKB1 increases spontaneous neurotransmitter release and asynchronous release in a calcium-dependent manner. **(A–D)** Conditional deletion of LKB1^F/F^ in cortical pyramidal neurons (E15 + 18 DIV) decreased mEPSC inter-event interval (inversely proportional to frequency) compared to control and this phenotype was rescued by acute application of EGTA-AM (Cre+EGTA-AM). LKB1-floxed mouse cortical neurons were infected by control and Cre viruses at 3–5 DIV and analyzed at 15–18 DIV. *n* = 3,032 events from 38 neurons for control, 3,159 events from 45 neurons for LKB1-deletion, 1,741 events from 21 neurons for EGTA-AM. *** *p* < 0.001, Mann-Whitney test. **(E–I)** Following single AP stimulation, LKB1-deficient cortical neurons showed decreased eEPSC amplitudes, and increased charge transfer and decay constant. **(E and F)** Colored trace shows averaged of 10 representative eEPSC traces (grey). *n* = 26 neurons for control, 29 neurons for LKB1-deficient neurons. *** *p* < 0.001, * *p* < 0.05, Mann-Whitney test. **(G–I)** Quantification of eEPSC in control (no Cre) versus LKB1-deficient cortical neurons (+Cre) showed decreased peak amplitude **(G)**, increased total charge transfer due to increased delayed release **(H)** and increased slow τ component of asynchronous release **(I)**. Individual values are available in **S1 Data**.

First, we measured mEPSC rates (**Fig 6A–6D**). Loss of LKB1 significantly increased mEPSC frequency, reflected by 25% decrease in inter-event interval compared to control (**Fig 6A, 6B and 6D**). This effect was reversed following brief incubation with a low-affinity, membrane-permeable, calcium chelator EGTA-AM (100 μM for 3–5 min) (**Fig 6C and 6D**), suggesting that the increased frequency of spontaneous release observed in LKB1-deficient axons is linked to accumulation of [Ca^2+^]_c_ rather than an increase in synapse numbers.

Next, we examined the properties of evoked EPSCs that were elicited by single AP. LKB1-deficient neurons show a 60% reduction in EPSCs amplitude (**Fig 6F**) compared to control (**Fig 6E**), suggesting reduced efficacy of fast, synchronous mode of neurotransmitter release (**Fig 6G**). In addition, LKB1-null neurons displayed increased spontaneous burst firing frequency (**S11 Fig**), and this defect can also cause decreased amplitude by reduction of the size of synaptic vesicle pools. Furthermore, the average amplitude of mEPSC was not altered in LKB1-deficient condition (**S10 Fig**), suggesting that neurotransmitter content of presynaptic vesicle does not cause this defect. Detailed analysis of isolated EPSC traces revealed that LKB1-null cortical neurons show prolonged form of delayed exocytosis (**Fig 6E and 6F**). This mode of asynchronous neurotransmitter release is mediated by a slow calcium sensor, and only becomes pronounced during presynaptic [Ca^2+^]_c_ accumulation [48,50–53]. Loss of LKB1 also leads to a significant increase of total charge transfer (**Fig 6H**) and more than 3-fold increase of slow tau constant (**Fig 6I**), indicating that asynchronous release was strongly potentiated in LKB1-null compared to control cortical neurons.

### Aberrant Presynaptic Ca^2+^ Homeostasis in LKB1-Deficient Axons Leads to Defective Short-Term Synaptic Plasticity

**Fig 7:**
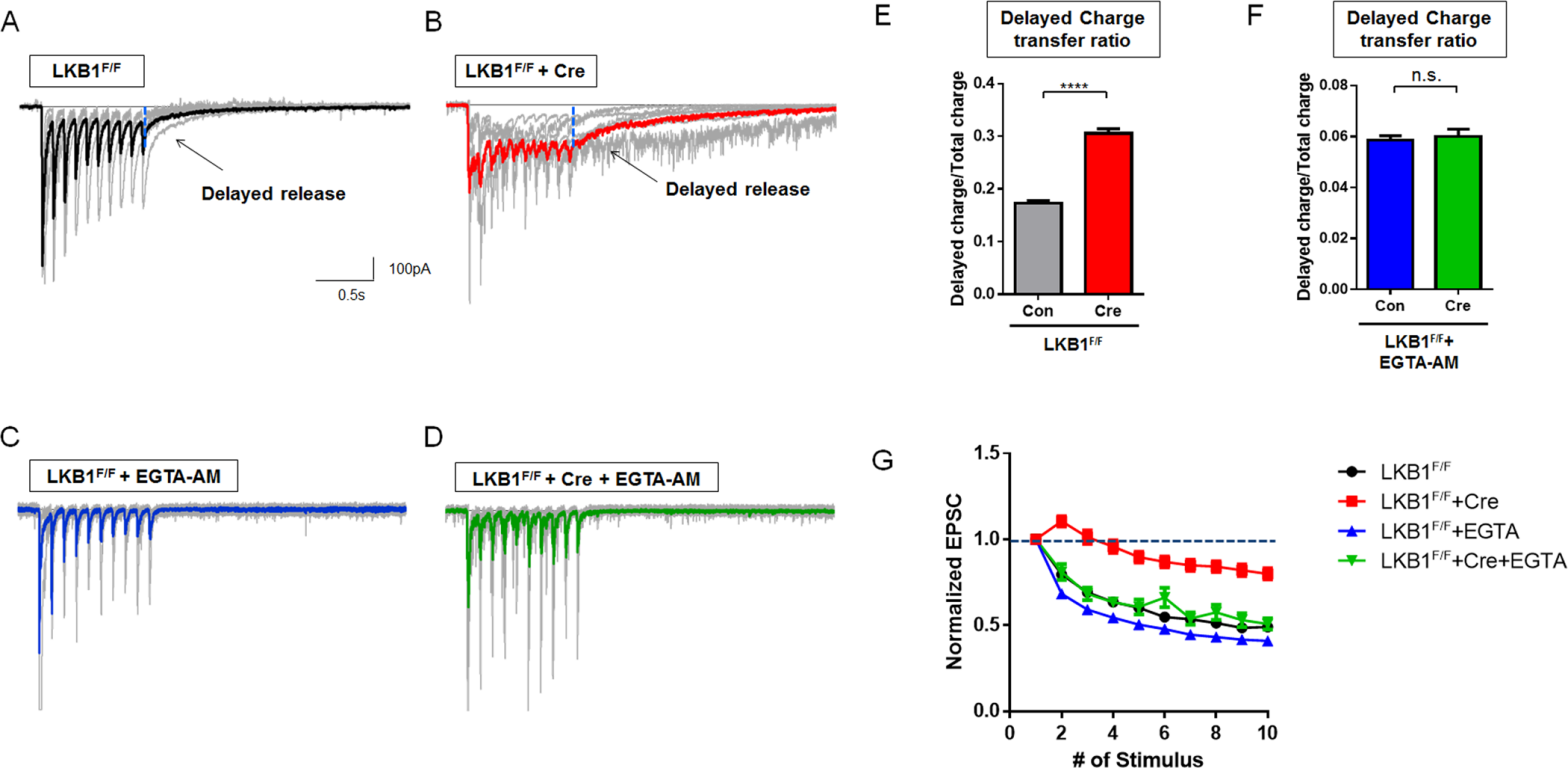
Conditional LKB1 deletion in cortical neurons abolishes short-term depression and increases asynchronous neurotransmitter release in a calcium-dependent manner. **(A–D)** Conditional deletion of LKB1^F/F^ cortical neurons was achieved by infection at 3–4 DIV with lentiviruses expressing Cre recombinase (B) or control only (A) followed by whole-cell patch-clamp recording in voltage-clamp mode at 15–18 DIV. LKB1-deficient cortical pyramidal neurons leads to abrogation of short-term depression of neurotransmitter release following repetitive stimulation (10 AP at 10 Hz), which was rescued by application of cell permeant calcium chelator (100 μM EGTA-AM 10 min. prior to recording; C, D). **(E and F)** Increased delayed release (measured after last AP during stimulation) in LKB1-deficient cortical neurons (E) was attenuated by EGTA-AM incubation (F). **(G)** Normalized eEPSC amplitudes plot following each AP stimulation during 10 Hz shows impaired short-term depression of evoked neurotransmitter release in LKB1-null neurons (red curve) compared to control neurons (black). Addition of EGTA-AM restored the short-term depression phenotype of LKB1-deficient neurons (green) back to control levels (blue). *n* = 321 responses from 22 neurons for control, 334 responses from 28 neurons for LKB1-deletion, 212 responses from 25 neurons for control+EGTA-AM, 90 responses from 8 neurons for LKB1-deletion+EGTA-AM. **** *p* < 0.0001, Mann-Whitney test. Individual values are available in **S1 Data**.

During repetitive high-frequency stimulation, central synapses undergo short-term synaptic plasticity expressed as facilitation/depression of synchronous EPSCs, which reflects progressive depletion of the readily releasable pool and recruitment of synaptic vesicles from the reserve pool. In addition, strong presynaptic calcium buildup [50,51] augments asynchronous exocytosis [49]. To test if the defect in presynaptic Ca^2+^ homeostasis observed in LKB1-deficient neurons leads to a change in short-term presynaptic plasticity, we applied 10 APs at 10 Hz. Consistent with single AP results, LKB1-null neurons show increased delayed synaptic release following 10 APs with significantly longer decay time, which is reflected by an increased delayed charge to total charge transfer ratio compared to control (**Fig 7A, 7B and 7E**). This increased delayed synaptic release was eliminated by acute application of EGTA-AM (**Fig 7C, 7D and 7F**) demonstrating that it is largely the result of increased [Ca^2+^]_c_ [47]. Also, this increased [Ca^2+^]_c_ can induce desynchronization of release [48], and loss of LKB1 leads to increased variability of neurotransmitter release properties during stimulus trains (**S12 Fig**). As previously reported [54], cortical pyramidal neurons show presynaptic depression following 10 Hz stimulation (**Fig 7A, 7B and 7G**). In contrast, LKB1-deficient cortical neurons did not display presynaptic depression during repetitive stimulations, and this was completely reverted by EGTA-AM application (**Fig 7C, 7D and 7G**). These results suggest that loss of LKB1 increased asynchronous release and alters short-term presynaptic plasticity by regulating presynaptic calcium homeostasis.

### Restoring MCU Expression Level Rescues the Synaptic Transmission Defects in LKB1-Null Neurons

**Fig 8:**
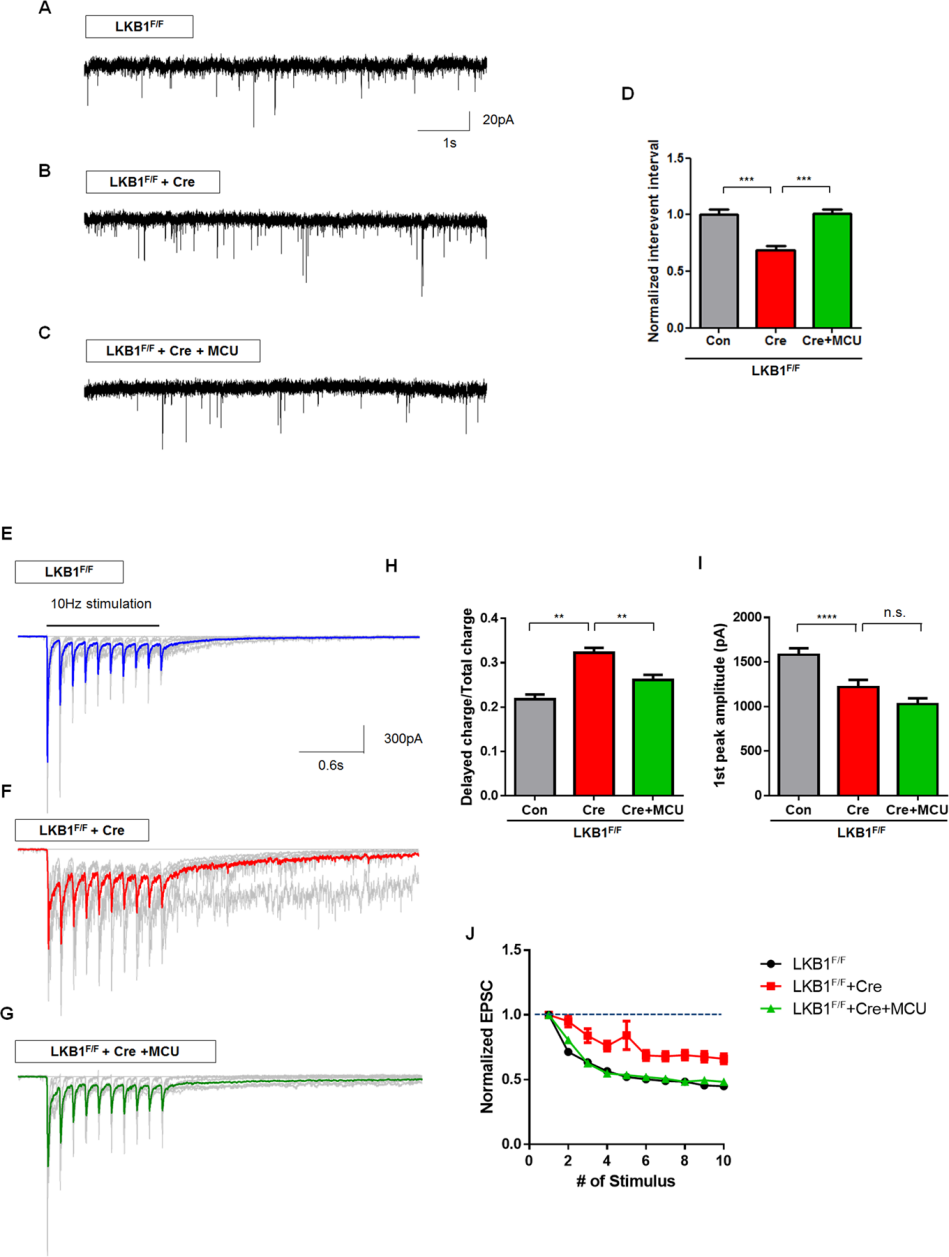
MCU overexpression restores presynaptic release properties in LKB1-deficient neurons. **(A–D)** MCU overexpression by lentiviral infection restored mEPSC frequency in LKB1-deficient cortical neurons. Values were normalized by the average of control from each experiment. *n* = 1,108 from 27 neurons for control, 1,030 events from 26 neurons for LKB1-deletion, 1,065 events from 21 neurons for LKB1-deletion+MCU. *** *p* < 0.001, Mann-Whitney test. **(E–J)** Colored representative traces show the average of nine traces (grey) of evoked EPSC (10 AP at 10 Hz). Increased delayed release observed LKB1-deficient neurons was partially rescued by MCU overexpression (**E–H**). Peak amplitude of first eEPSC was not rescued by MCU overexpression. (**I**). MCU overexpression restored short-term depression in LKB1-deleted neurons back to control levels (**J**). Graph represents normalized EPSC amplitude. *n* = 140 responses from 20 neurons for control, 169 responses from 17 neurons for LKB1-deletion, 160 responses from 17 neurons for LKB1-deletion+MCU. ** *p* < 0.01, **** *p* < 0.0001. Mann-Whitney test. Individual values are available in **S1 Data**.

We tested if there is a causal relationship between the defect in MCU-dependent [Ca^2+^]_c_ clearance observed in LKB1-null cortical axons and the abnormal neurotransmission properties described above (**Figs 6 and 7**). Remarkably, co-infection of LKB1^F/F^ dissociated cortical neuron cultures with MCU- and Cre-encoding lentiviruses rescued the increased frequency of spontaneous mEPSC to control levels (**Fig 8A–8D**). In addition, from the Ca^2+^ imaging, we observed significantly elevated basal level of presynaptic [Ca^2+^]_c_ compared to control, and this is also restored to control level following MCU overexpression (**S13 Fig**). Expression of MCU in LKB1-deficient neurons also reduced the delayed charge/total charge transfer ratio to control levels following the end of the 10 Hz stimulation (**Fig 8E–8H**). Decreased amplitude of the first evoked AP in LKB1-null neurons was not rescued by MCU overexpression (**Fig 8I**) suggesting that this change in amplitude of EPSC might involve a postsynaptic change or reduced synaptic vesicle pool size independent of MCU-dependent presynaptic Ca^2+^ clearance. Finally, following 10 Hz stimulation, MCU overexpression re-established short-term depression of neurotransmitter release in LKB1-deficient neurons back to control levels (**Fig 8J**).

## Discussion

Taken together, our results demonstrate that (1) presynaptic mitochondria control neurotransmitter release properties along the axon of cortical pyramidal neurons through their ability to regulate presynaptic Ca^2+^ clearance in a bouton-specific manner and (2) that the LKB1 pathway regulates the ability of mitochondria to regulate presynaptic Ca^2+^ homeostasis and neurotransmitter release properties. Our results uncover the critical role played by the presence or absence of mitochondria at individual boutons in differential control of Ca^2+^ homeostasis and exocytosis of SVs. LKB1 regulates presynaptic Ca^2+^ uptake into mitochondria through regulation of MCU abundance. In LKB1-null axons, alterations in mitochondria-dependent presynaptic Ca^2+^ homeostasis leads to drastic changes of neurotransmitter release properties including increased asynchronous release at the expense of fast, synchronous forms of neurotransmitter release as well as alterations of short-term presynaptic plasticity. We demonstrate that these alterations in neurotransmitter release properties are causally linked to down-regulation of MCU expression in LKB1-null axons since restoration of MCU expression levels rescues the presynaptic Ca^2+^ accumulation defects in LKB1-deficient axons and the vast majority of changes of neurotransmitter release properties. Our results reveal a new LKB1-dependent signaling mechanism regulating neurotransmitter release properties by controlling the ability of presynaptic mitochondria to uptake Ca^2+^ through MCU.

### Mitochondria Determine Bouton-Specific Presynaptic Release Properties via Ca^2+^ Clearance

As mentioned above, a single axon has spatial heterogeneity of presynaptic Ca^2+^ levels during AP-evoked synaptic transmission [6–8]. In addition, the same pyramidal neurons in the neocortex have target cell-specific neurotransmitter release properties [9,11,12]. Layer 2/3 pyramidal cells in the neocortex show distinct short-term synaptic plasticity at synapses made onto two types of GABAergic interneurons. Excitatory synapses made by axons of pyramidal neurons onto parvalbumin-positive interneurons (basket cells), display high level of Ca^2+^ influx upon single AP stimulation and high release probability, and short-term depression (STD). However, synapses made by the same axon of pyramidal cells onto somatostatin-positive (bitufted or Martinotti cells) display low Ca^2+^ influx upon single AP stimulation, low release probability and short-term facilitation (STF). Interestingly, this facilitation phenotype is abolished by EGTA injection to presynaptic cells, therefore the residual Ca^2+^ level is important to modulate the STF on these synapses [12].

However, some of other findings showed that the synapses of pyramidal-to-bitufted cells have lower single-AP evoked presynaptic Ca^2+^ levels, higher failure rates of synaptic transmission, and smaller EPSP amplitudes [9,12]. Our data using syp-pHluorin imaging of neurotransmitter release demonstrate that presynaptic boutons associated with mitochondria display lower level Ca^2+^ influx during evoked neurotransmission and lower neurotransmitter release compared to presynaptic boutons not associated with mitochondria. Our data are normalized for the size of the SV pool (using de-acidification by NH_4_Cl; see for 32, 34, 36), therefore, we cannot infer if some of these differences reflect a change in the size of the presynaptic vesicle pool (sum of the readily releasable pool [RRP] and the reserve pool [RP]). In fact, non-normalized signals showed no difference in evoked syp-pHluorin signals between mitochondria-free boutons and boutons associated with mitochondria. This is highly reminiscent of a previous report, which did not use total pool normalization although the study elucidated that mitochondria contribute to the variability of presynaptic strength bouton-specifically [55]. Total vesicle pool size of mitochondria-free presynapses measured by syp-pHluorin de-acidification is smaller than mitochondria-associated ones (**S4 Fig**). In addition, a recent electron microscopy (EM) study described that presynaptic sites associated with mitochondria have a significantly larger number of SV compared to presynaptic boutons not associated with mitochondria [56]. Therefore, synaptic transmission could be larger at presynaptic boutons associated with mitochondria because they possess larger RRP. However, several reports showed that independent of RRP size, presynaptic Ca^2+^ level affects Pv (fusion probability of individual vesicle upon single AP) [57,58]. Overall, our study implies that a single neuron displays significantly different release properties through bouton-specific association with mitochondria and therefore that mitochondria represent a key effector of synapse-specific neurotransmitter release properties, including short-term facilitation versus depression, through their ability to regulate Ca^2+^ clearance.

### Mitochondria as Key Regulators of Presynaptic Ca^2+^ Homeostasis

Multiple molecular mechanisms involved in Ca^2+^ clearance have been postulated to operate at presynaptic sites such as plasma membrane Ca^2+^-ATPase (PMCA), Na^+^/Ca^2+^ exchanger (NCX), smooth endoplasmic reticulum Ca^2+^-ATPase (SERCA), and MCU [17,19,22,59]. Conflicting evidence has been reported regarding the importance of mitochondria-dependent presynaptic Ca^2+^ clearance in the regulation of presynaptic release properties in various types of axons such as the neuromuscular junctions (NMJ) and Calyx of Held [17,19]. Blockade of mitochondrial Ca^2+^ sequestration by mitochondrial depolarization or inhibition of MCU increased presynaptic [Ca^2+^] or decay time, but induced depression of presynaptic release or slowed recovery from depression [17,19]. In addition, recent report supports mitochondria Ca^2+^ level in resting state modulates spontaneous mEPSC frequency [60]. In retinal bipolar neurons and *Drosophila* NMJ, mitochondria have limited effects in presynaptic Ca^2+^ handling during PMCA inhibition or long stimulation [20,61]. In rat hippocampal CA3 region, PMCA isoform 2a is localized mainly at excitatory presynaptic sites, and pharmacological blockage of PMCA increased mEPSC frequency and paired-pulse facilitation (PPF) of evoked EPSCs [59]. SERCA pump also contributes presynaptic Ca^2+^ clearance and inhibition of this pump induces PPF at the frog NMJ and rat hippocampal CA1 region [62,63]. These divergent results might reflect that specific types of synapses might utilize different molecular effectors to clear presynaptic Ca^2+^ because of vast differences in the cytoplasmic volume at these different presynaptic terminals and also because these different type of Ca^2+^ clearance mechanisms vary greatly in their spatial and temporal dynamics. Nevertheless, our results reveal that mitochondria represents a critical component of presynaptic Ca^2+^ homeostasis in cortical axons and as such play a critical role in regulating neurotransmitter release properties in a synapse-specific way.

### Mitochondrial Ca^2+^ Uptake Regulates ATP Synthesis

Several studies have suggested that mitochondrial Ca^2+^ import leads to increased ATP production [15,64]. Several mitochondrial matrix dehydrogenases are directly or indirectly activated by Ca^2+^ present in the mitochondrial matrix. Activation of these proteins increases NADH levels, which drives electron transfer, ultimately resulting in increased ATP production by the ATP synthase. In addition, mitochondrial Ca^2+^ can stimulate ATP production by inducing metabolite transport through the aspartate/glutamate exchanger that contains an EF-hand, Ca^2+^-binding domain. In *Drosophila* motor neurons, presynaptic terminals have increased mitochondrial matrix pH (pH_m_), membrane potential (Δψ_m_), and NADH level, which can lead to ATP production following AP triggering [65]. More recent evidence demonstrated that in rat hippocampal neurons, the main driver of ATP consumption is actually presynaptic release and synaptic vesicle recycling [66]. Therefore, the LKB1 signaling pathway may also coordinate ATP production by regulating mitochondrial Ca^2+^ influx which is directly triggered by cytoplasmic Ca^2+^ increase during action potential invasion of the presynaptic terminal and opening of voltage-gated Ca^2+^ channels. However, our results also suggest that the presence or absence of mitochondria at individual presynaptic sites cannot simply explain differences in ATP demand upon neurotransmitter release. In fact, our results suggest that the presynaptic boutons associated with mitochondria show lower levels of neurotransmitter release than boutons not associated with mitochondria. Our data demonstrate that the main function of presynaptic mitochondria in axons of pyramidal neurons is to regulate presynaptic Ca^2+^ dynamics and neurotransmitter release properties in a synapse-specific manner.

### LKB1 Regulates MCU Expression and the Ability of Presynaptic Mitochondria to Uptake Ca^2+^

Very little is currently known about signaling pathways regulating MCU expression and function in mitochondria, but recent reports suggested that in cardiomyocytes, MCU can be regulated by CAMK2-dependent phosphorylation, which is activated during ischemic injury [67]. Using a model of neuronal excitotoxicity where neurons are exposed to high doses of NMDA, a recent study has shown that MCU is down-regulated at the transcriptional level in a CAMK2-Npas4-dependent manner, leading the authors to hypothesize that MCU expression is activity-dependent and participates in a homeostatic mechanism underlying neuroprotection [68]. However, our preliminary results suggest that MCU mRNA abundance is unchanged in LKB1-deficient neurons compared to control (**S14 Fig**). Another hypothesis could be that LKB1 regulates the translation, protein stability and/or the import of MCU protein into mitochondria. MCU, like >99% of mitochondrial proteins, is genomically encoded, and previous work shows that mitochondrial protein import can be regulated through phosphorylation by cytosolic kinases [69]. Future work will need to address these possibilities.

Overall, our results identify a new function for the serine/threonine kinase LKB1 in the regulation of presynaptic Ca^2+^ homeostasis through MCU-dependent presynaptic Ca^2+^ uptake into mitochondria. This study uncovers the critical importance of presynaptic mitochondrial capture for the regulation of neurotransmitter release properties. Our finding also has important implications for microcircuit properties. As described above, it is possible that target cell type-specific plasticity could be influenced by the presence or absence of mitochondria presynaptically. Recent studies showed that, in hippocampus, Elfn1, a synaptic cell adhesion molecule containing extracellular leucine-rich repeat domain, is selectively expressed at somatostatin-positive interneurons, and induces STF in retrograde [70,71]. Therefore, these synaptic cell adhesion molecules could be potential determinants of mitochondria capture in a postsynaptic target cell type-dependent way. Future experiments will be needed to explore this hypothesis.

## Materials and Methods

### Animals

All animal experiments employed the protocols approved by Institutional Animal Care and Use Committee (IACUC) as well as NIH guidelines at The Scripps Research Institute, CA, and Columbia University, NY (Protocol#AC-AAAH4600). All mice were maintained in a 12 h light/dark cycle. C57BI/6J, Balb/C were used for dissociated cortical culture, and floxed LKB1 (Stk11^tm1.1Rdp^) and Nex^Cre^ mice were described previously [28].

### Plasmids

We inserted the cDNA encoding Cre-recombinase to pCAG-IRES-GFP (pCIG2 [72]) by replacing IRES-GFP, and we used pCAX as a control to Cre-expressing vector. We conjugated mitochondrial targeting sequences from human cytochrome c oxidase subunit VIII to mTagBFP, mCherry and GCaMP5G (from pCMV-GCaMP5G; Douglas Kim and Loren Looger, Addgene #31788) by swapping DsRED1 in pCAG-mitoDsRED1 [28]. pCAG:vGlut1-mCherry and pCAG:vGlut1-GCaMP5G were created by fusion of rat vGlut1 and mCherry or GCaMP5G. Human MCU (gift from Mark Anderson [67]) cDNA was amplified with C-terminal HA- epitope tag and subcloned into pCAG plasmid. Synaptophysin-pHluorin [31] was subcloned into pCAG vector. Synapsin promoter driven lentiviral shuttle vectors, pSyn-Cre and pSyn-ΔCre, were provided by Anton Maximov laboratory.

### Primary Neuronal Culture

Embryonic mouse cortices (E15.5) were dissected in Hank’s Buffered Salt Solution (HBSS) supplemented with HEPES (10 mM, pH 7.4), and incubated in HBSS containing papain (Worthington; 14 U/ml) and DNase I (100 μg/ml) for 20 min at 37°C. Then, samples were washed with HBSS, and dissociated by pipetting. Cell suspension was plated on poly-D-lysine (1 mg/ml, Sigma)-coated glass bottom dishes (MatTek) or coverslips (BD bioscience) in Neurobasal media (Invitrogen) containing B27, Glutamax, FBS (2.5%) and penicillin/streptomycin (all supplements were from Invitrogen). After 5 to 7 d, media were changed with FBS-free supplemented Neurobasal media.

### Ex Utero Cortical Electroporation

We injected plasmids and 0.5% Fast Green (Sigma, 1:20 dilution) mixture using a micro-injector (Picospritzer III, Parker) into the lateral ventricles of isolated head of E15.5 mouse embryo, and electoporated using an electroporator (ECM 830, BTX) with five pulses of 20 V at 100 ms interval. Then, we followed the dissociated neuronal culture protocol as previously described.

### Western Blot and Antibodies

Hippocampal regions were dissected from 3-wk-old wild-type (NEX^Cre^;LKB1^F/+^ or NEX^Cre^;LKB1^+/+^) or Nex^Cre^;LKB1^F/F^ conditional knockout (KO) mice and lysed with RIPA buffer containing 1% NP-40, 0.5% sodium deoxycholate, 0.1% SDS, 150 mM NaCl in 50 mM Tris buffer (pH 8.0) supplemented with the cocktail of protease inhibitors (Roche). Samples were loaded onto SDS-PAGE gels and transferred to polyvinylidene difluoride (PVDF) membrane (Amersham). After transfer, membranes were blocked for 30 min with blocking buffer containing 5% skim milk in TBS-T (20 mM Tris-HCl [pH7.4], 150 mM NaCl, 0.1% Tween 20). Then, primary antibodies were incubated overnight at 4°C, and washed three times by TBS-T. HRP-coupled secondary antibodies (Invitrogen) were incubated for 1 h at room temperature followed by three times washes with TBS-T. Chemiluminescence images were taken by Fluorochem Q imager (ProteinSimple) and quantified using AlphaView software (ProteinSimple). Primary antibodies are rabbit anti-MCU (1:2000, Sigma), mouse anti-OxPhos complex kit (1:2000, Invitrogen), rabbit anti-LKB1 (1:1000, Cell Signaling), and mouse anti-actin (1:5000, Millipore).

### RT-qPCR

RNA was isolated from hippocampi of P19 Control and Nex-Cre;LKB1^F/F^ mice with NucleoSpin RNA (MACHEREY-NAGEL) according to the instructions of the manufacturer. cDNA was reverse transcribed using SuperScript III First-Strand Synthesis SuperMix for qRT-PCR (Life Technologies) according to the directions of the manufacturer. The resulting cDNA was analyzed using quantitative PCR with Power SYBR Green PCR Master Mix (Life Technologies) in an Eppendorf Realplex using the following primers:

Actin (5′- GGCTGTATTCCCCTCCATCG -3′, 5′- CCAGTTGGTAACAATGCCATGT-3′)

MCU (5′-GAGCCGCATATTGCAGTACG-3′, 5′-CGAGAGGGTAGCCTCACAGAT-3′)

### Live Imaging

Transfected cortical neurons were imaged at 15-18DIV with EMCCD camera (Andor, iXon3-897) on an inverted Nikon Ti-E microscope (40x objective NA0.6 with 1.5x digital zoom or 60x objective NA1.4); 488 nm and 561 nm lasers shuttered by Acousto-Optic Tunable Filters (AOTF) or 470 nm, 555 nm, and 360 nm Spectra X LED lights (Lumencor) were used for the light source, and a custom quad-band excitation/mirror/emission cube (based off Chroma, 89400) followed by clean up filters (Chroma, ET525/50, ET600/50, ET435/26) were applied for excitation and emission. We used the modified normal tyrode solution as a bath solution at 37°C, which contained (in mM): 145 NaCl, 2.5 KCl, 10 HEPES pH 7.4, 1.25 NaH_2_PO_4_, 4 CaCl_2_, 10 glucose. For presynaptic calcium imaging during spontaneous release, we added picrotoxin (50 μM, Tocris) and images were captured by 1 s interval for 10 min. For calcium imaging on evoked release, we added APV (50 μM, Tocris) and CNQX (20 μM, Tocris) in bath solution. For testing MCU inhibitor effect, Ru360 (10 μM, EMD Millipore) was incubated for 3 min before imaging. Evoked releases were triggered by 1 ms current injections with a concentric bipolar electrode (FHC) placed 20 μm away from transfected axons. We applied 20 AP or 100 AP at 10 Hz with 20 V using the stimulator (Model 2100, A-M systems) and imaged with 350 ms interval (2.86 Hz) during 90 s for vGlut1-GCaMP5G signals and 1 s interval (1 Hz) for 210 s for mito-GCaMP5G signals. At the end of experiments, we added the calcium ionophore ionomycin (5 μM, EMD Millipore) and continued imaging with 1 s interval to obtain Fmax value for each axon segment imaged. For syp-pHluorin imaging, 20 AP or 100 AP were applied at 10 Hz and neurons were imaged with 500 ms interval (2 Hz) during 90 s, then the bath solution was changed with tyrode solution containing 55 mM NH_4_Cl for Fmax value. Images were analyzed in Fiji (Image J) using a Time Series Analyzer (v3.0) plugin. Single presynaptic bouton imaged from multiple experiments were analyzed individually and pooled for statistical analysis. Each vGlut1-GCaMP5G, mito-GCaMP5G or syp-pHluorin puncta and nearby backgrounds were selected by circular ROIs and intensities were measured by plug-in. After intensities were corrected for background subtraction, ΔF values were calculated from (F-F0). F0 values were defined by averaging 10 frames before stimulation, and F_max_ values were determined by averaging 10 frames of maximum plateau values following ionomycin or NH_4_Cl application, then, used for normalization. Mito-free presynapses for Ca^2+^ and pHluorin imaging were defined by 3.5 μm distance from axonal mitochondria.

### Statistical Analysis

All graphs were drawn using Prism (GraphPad Software) and statistical analyses were performed with the same software. We used non-parametric tests (Mann-Whitney test) when at least one of the group tested displayed a distribution that deviated significantly from normality. For comparing before and after drug treatment, we employed paired *t* test (**Fig 3A–3C**). Statistical tests and significance levels are indicated in figure legends.

### Lentiviral Production and Infection

Recombinant lentiviruses were produced from human embryonic kidney 293T (HEK293T) cells by co-transfection with shuttle vectors, LP1, LP2, and VSV-G. FuGENE transfection reagent (Roche) was used and 24 h after transfection, media were exchanged with Neurobasal media, and 48hrs later, supernatants were harvested and centrifuged for removing cellular debris. Then, 200 μl of viral supernatants was added to each 24-well containing dissociated cortical neurons at 3–5 DIV and recorded at DIV 15–18 [73]. For MCU rescue experiments, because of apoptotic effect by mitochondrial calcium increase, we only added 10 μl supernatants.

### Electrophysiology

Analysis of synaptic transmission in cultured neurons was performed as described [74,75]. Evoked synaptic responses were triggered by 1 ms current injections with a local extracellular stimulating electrode (FHC, Inc.), and were monitored from randomly selected nearby neurons by whole-cell patch clamp recordings using a Multiclamp 700B amplifier in voltage clamp mode (Axon Instruments, Inc.). Short-term presynaptic plasticity was assessed by stimulating at 10 Hz for 1 s. The frequency, duration, and magnitude of extracellular stimuli were controlled with Model 2100 Isolated Pulse Stimulator (A-M Systems, Inc.). The whole-cell pipette solution contained 135 mM CsCl_2_, 10 mM HEPES-NaOH pH 7.4, 1 mM EGTA, 1 mM Na-ATP, 0.4 mM Na-GTP, and 1 mM QX-314. The resistance of filled pipettes varied between 3–5 mOhm. The bath solution contained 140 mM NaCl, 5 mM KCl, 2 mM CaCl_2_, 0.8 mM MgCl_2_, 10 mM HEPES-NaOH pH 7.4, and 10 mM glucose. EPSCs were isolated pharmacologically by addition of 100 μM picrotoxin to the bath solution. For calcium chelation, 100 μM EGTA-AM was added 10 min before recording. The currents were sampled at 10 kHz and analyzed offline using pClamp10 (Axon Instruments, Inc.) software. For illustration purposes only, traces of evoked synaptic currents were filtered at 50 Hz.

## Supporting Information

S1 Data List of all individual data values obtained for quantifications corresponding to each figure panel. (XLSX)

S1 Fig Imaging of presynaptic and mitochondrial Ca^2+^ dynamics with GCaMP5G fusion proteins in cortical axons. **(A and C)** Ca^2+^ dynamics at presynaptic sites occupied by mitochondria were monitored using expression of vGlut1-GCaMP5G and mito-mCherry in axons of cortical layer 2/3 neurons following ex utero electroporation at E15.5 followed by dissociation and culture for 15–17 DIV. **(C)** Representative kymograph of vGlut1-GCaMP5G (green) and mito-mCherry (red) before, during and after imposing 100 AP at 10 Hz (vertical white bar). **(B and D)** Calcium dynamics inside the matrix of mitochondria localized at a presynaptic bouton were measured using expression of mito-GCaMP5G and vGlut1-mCherry at the same condition. **(B)** Representative kymograph of mito-GCaMP5G (green) and vGlut1-mCherry (red) before, during and after imposing 100 AP at 10 Hz (white bar). **(E)** Intramitochondrial calcium influx shows significantly slower decay time than presynaptic Ca^2+^ clearance. Individual values are available in **S1 Data**.
(TIF)

S2 Fig AP-dependent responses of cytosolic GCaMP5G and vGlut1-GCaMP5G at presynaptic sites. **(A and B)** vGlut1-GCaMP5G is spatially concentrated at presynaptic sites. **(C-H)** Cytosolic GCaMP5G and vGlut1-GCaMP5G signals were obtained by 1 s stimulation at different frequencies for given APs. Peak values of cytosolic GCaMP5G signals reached a maximum response at 20 AP, but vGlut1-GCaMP5G signals reached a maximum response at 50 AP, and total Ca^2+^ amount was reached maximum from 50 AP for cytosolic GCaMP5G, but from 80 AP for vGlut1-GCaMP5G. *n* = 14 for cyto-GCaMP5G, 12 for vGlut1-GCaMP5G. ** *p* < 0.01, *** *p* < 0.001. Mann-Whitney test. Individual values are available in **S1 Data**.
(TIF)

S3 Fig Mitochondria-free presynaptic boutons have increased cytoplasmic Ca^2+^ under various stimulation conditions. Presynaptic [Ca^2+^]_c_ dynamics was measured at mitochondria-associated and mitochondria-free boutons using vGlut1-GCaMP5G and mito-mTagBFP in axons of cultured cortical neurons following ex utero electroporation at E15.5 and imaged at 15–17 DIV. Mitochondria-free boutons show significantly increased normalized peak values and total charge transfer (area under curve) during repetitive stimulation (10 AP, 20 AP, 50 AP, and 100 AP at 10 Hz). 10 AP: *n* = 62 for mito, and 43 for mito-free from 14 neurons. 20 AP: *n* = 62 for mito, and 40 for mito-free from 14 neurons. 50 AP: *n* = 47 for mito, and 30 for mito-free from 10 neurons. * *p* < 0.05 and ** *p* < 0.01, Mann-Whitney test. Individual values are available in **S1 Data**.
(TIF)

S4 Fig Mitochondria-free presynaptic boutons have larger total SV pool size and non-normalized syp-pHluorin signals show no difference. **(A and B)** Fmax values of syp-pHluorin was obtained by NH_4_Cl (50 mM) incubation, and both Fmax and Fmax-F0 values were significantly lower at mito-free than mitochondria-associated boutons. *n* = 26 for mitochondria-associated boutons and 15 for mitochondria-free boutons from 15 neurons in 20 AP condition. *n* = 31 for mitochondria-associated boutons and 17 for mitochondria-free boutons from 15 neurons in 100 AP condition. * *p* < 0.05, ** *p* < 0.01 Mann-Whitney test. Individual values are available in **S1 Data**.
(TIF)

S5 Fig LKB1-deficient neuronal mitochondria are normally captured at presynaptic sites in mature cortical neurons. **(A and B)** Time-lapse microscopy of cortical neurons in culture reveals that mitochondria (labeled by Mito-mCherry (red)) occupy the same percentage of presynaptic boutons (vGlut1-Venus (green)) in WT and LKB1-mutant neurons at mature stage (E15.5+17DIV). Overlapping pixels maps shown in A and B were created in Fiji/ImageJ using the Colocalization Threshold plugin. **(C)** There was no significant difference in the percentage of stationary mitochondria between control and LKB1-deficient neurons. *n* = 24 for control, 25 for LKB1-deletion. **(D and E)** The percentage of vGlut1 puncta occupied by mitochondria as well as mitochondria captured at presynaptic sites was not altered in mature LKB1-deficient axons. *n* = 17 for control, 18 for LKB1-deletion. **(F and G)** Mitochondria and vGlut1 puncta density was also not altered in mature LKB1-deificient axons. *n* = 17 for control, 18 for LKB1-deletion. Mann-Whitney test. Individual values are available in **S1 Data**.
(TIF)

S6 Fig Presynaptic differentiation is not altered in LKB1-deficient axons in vitro and in vivo. **(A and B)** HA-Neuroligin-1-expressing COS7 cells (blue) were co-cultured with WT and LKB1-null cortical neurons following ex utero electroporation at E15.5 and cultured for 9-12DIV. Single axons and their presynaptic boutons were visualized in cortical neurons by ex utero co-electroporation of mCherry and vGlut1-Venus by electroporation. Scale bar = 20 μm. **(C)** Quantification of the linear density of vGlut1-Venus-positive presynaptic boutons (#boutons/10 microns of axons growing over cells). Both WT and LKB1-null axons showed similar increased density of vGlut1-Venus puncta around neuroligin-1-expressing COS7 cells compared to cells transfected with CD8 (control). **(D)** Quantification of vGlut1-Venus-positive presynaptic bouton area formed over COS7 transfected with the indicated plasmids. Both WT and LKB1-null neurons had increased vGlut1-Venus area on the neuroligin-1-expressing COS7 cells. *n* = 16 for CD8 on WT, 16 for NL-1 on WT, and 16 for NL-1 on KO. ** *p* < 0.01, *** *p* < 0.001. Kruskal-Wallis test. **(E)** Plasmids encoding Cre recombinase, HA-mCherry and Flex::Synaptophysin-Venus were co-expressed by in utero cortical electroporation of mouse embryos at E15.5 (harvested at P16). Only Cre-expressing neurons could express synaptophysin-Venus signals following Cre-mediated inversion. LKB1-null neurons (right panels) displayed reduced density of synaptophysin-Venus staining compared to control (left panels) which is probably due to reduced terminal axon branching in ipsilateral layers 2/3 and 5, as previously reported [28]. Scale bar = 150 μm. **(F and G)** In high-magnification images of axon segments in hemisphere contralateral to the electroporation side, the linear density of synaptophysin-Venus presynaptic boutons was not altered in LKB1-deficient neurons compared to control. Scale bar = 30 μm. **(H)** Quantification of linear density of synaptophysin-Venus puncta (#boutons/microns). Total cumulative length of axon quantified = 2,831μm from 3 pups for control, 2,613 μm from 3 pups for LKB1-deletion. Mann-Whitney test. Individual values are available in **S1 Data**.
(TIF)

S7 Fig Overexpressed MCU-HA is correctly targeted to mitochondria in cortical axons. (**A–C**) Expression of HA-tagged MCU in cortical layer 2/3 neurons (ex utero electroporation, dissociated at E15.5 and cultured for 5 DIV) shows punctate distribution along the axon (MAP2-negative; arrowheads in A and B). **(D–F)** Co-expression of MCU-HA and Mito-mCherry in cortical layer 2/3 neurons (ex utero electroporation and dissociated at E15.5 + 5 DIV) reveals the co-localization of MCU with mitochondria throughout the neurons including the axon (arrowheads in D and E). Scale bar = 40 μm.
(TIF)

S8 Fig LKB1-null axons show increased presynaptic Ca^2+^ level and half-decay time during spontaneous network activity. **(A–D)** Increased maximal presynaptic Ca^2+^ accumulation and delayed calcium clearance during spontaneous neurotransmitter release in layer 2/3 cortical pyramidal neurons. **(A)** Captured images from vGlut1-GCaMP5G timelapse series at presynaptic sites associated with mitochondria (mito-mCherry signal not shown). **(B–D)** Analysis of the temporal dynamics of vGlut1-GCaMP5G signals during spontaneous release (B) shows increased half-decay time (C) and increased maximum intensity (D) of [Ca^2+^]_c_ in LKB1-deficient neurons (red) compared to control (gray). *n* = 35 for control from 12 neurons, 33 for LKB1–null from 11 neurons. *** *p* < 0.001 and ** *p* < 0.01, Mann-Whitney test. Scale bar = 10 μm. Individual values are available in **S1 Data**.
(TIF)

S9 Fig LKB1 kinase-dead mutants cannot rescue presynaptic Ca^2+^ clearance in LKB1-deficient neurons. **(A)** Expression of wild-type LKB1 restores the elevated presynaptic Ca^2+^ observed in LKB1-deficient neurons back to control level. LKB1^F/F^ cortical neurons were ex utero electroporated with control, Cre, and LKB1-WT (E15.5 + 16–17 DIV). **(B)** LKB1 kinase mutants, D194A and K78A, were not able to rescue calcium clearance defects on LKB1-null axons. **(C)** Total charge transfer (total area under curve) is analyzed from A and B. *n* = 32 for control, 36 for LKB1-deletion, 32 for LKB1-WT, 33 for LKB1-DA, 35 for LKB1-KA. * *p* < 0.05, ** *p* < 0.01, Mann-Whitney test. Individual values are available in **S1 Data**.
(TIF)

S10 Fig mEPSC amplitude of LKB1-null neurons is not altered. The amplitude of mEPSC measured by patch-clamp in LKB1-null neurons display non-significant difference compared to WT neurons. *n* = 4,012 events from 21 neurons for control and 4,401 events from 18 neurons for LKB1-null. Mann-Whitney test. Individual values are available in **S1 Data**.
(TIF)

S11 Fig LKB1-null neuronal networks in culture show increased burst firing frequency and increased burst duration, but decreased amplitude. **(A and B)** LKB1-null neurons show more frequent burst firing but smaller amplitude. Dissociated cultures of LKB1^F/F^ cortical neurons were infected with control or Cre-expressing lentivirus at 3–5 DIV and recorded at 15–18 DIV. **(C and D)** Analysis of single bursts reveals that LKB1-deficient neurons display longer burst width/duration, but similar charge transfer compared to control cortical neurons. **(E–H)** Quantification of burst frequency, width, amplitude and charge transfer. Data from each experiment were normalized by average of control values. *n* = 24 neurons for control, 29 for LKB1-deletion. ** *p* < 0.01, **** *p* < 0.0001, Mann-Whitney test. Individual values are available in **S1 Data**.
(TIF)

S12 Fig Desynchronization of EPSC is increased in LKB1-null neurons compared to control. **(A and B)** Time-to-peak values from each EPSC peaks during 10 AP stimulation (10 Hz) were normalized by first response and plotted. **(C)** Standard deviation (SD) values of individual EPSCs are plotted from repetitive recordings (7–15) of each cortical neuron. Pooled data were analyzed with Mann-Whitney test. ** *p* < 0.01, *** *p* < 0.001, **** *p* < 0.0001. *n* = 28 for control, 27 for LKB1-deficient. Individual values are available in **S1 Data**.
(TIF)

S13 Fig Increased basal level of presynaptic Ca^2+^ in LKB1-deficient neurons is rescued by MCU overexpression. Basal presynaptic [Ca^2+^]_c_ levels were monitored using vGlut1-GCaMP5G in LKB1-null and MCU-overexpressing LKB1-null axons at 15–17 DIV. Basal level of presynaptic [Ca^2+^]_c_ is significantly elevated in LKB1-null axons compared to control cortical neurons, and MCU overexpression restores the increased basal Ca^2+^ to control levels. * *p* < 0.05, ** *p* < 0.01. Mann-Whitney test. *n* = 22 for control, 18 for LKB1-deficient, and 18 for LKB1-deficient + MCU. Individual values are available in **S1 Data**.
(TIF)

S14 Fig MCU mRNA level is not altered in LKB1-null hippocampi. MCU mRNA was extracted and measured by RT-qPCR. Values were normalized by β-actin mRNA level. MCU mRNA level was not significantly changed in LKB1 knockout hippocampi (Nex-Cre;LKB1^F/F^, P19). Unpaired *t* test. Individual values are available in **S1 Data**.
(TIF)

## Abbreviations

AP: action potentia
CNS: central nervous system
COXIII: Subunit III of cytochrome c oxidase
DIV: days in vitro
KO: knockout
LKB1: Liver Kinase B1
MCU: Mitochondrial Calcium Uniporter
mEPSC: miniature Excitatory Post-Synaptic Current
NMJ: neuromuscular junctions
PMCA: plasma membrane Ca2+-ATPase
RP: reserve pool
RRP: readily releasable pool
SERCA: smooth endoplasmic reticulum Ca2+-ATPase
STD: short-term depression
STF: short-term facilitation
VDAC: voltage-dependent anion channel
VGCC: voltage-gated Ca2+ channels
vGlut1: vesicular glutamate transporter 1
WT: wild type
