# Correction: LKB1 Regulates Mitochondria-Dependent Presynaptic Calcium Clearance and Neurotransmitter Release Properties at Excitatory Synapses along Cortical Axons

**DOI:** 10.1371/journal.pbio.3000040

**Published:** 2018-09-26

**Authors:** Seok-Kyu Kwon, Richard Sando, Tommy L. Lewis, Yusuke Hirabayashi, Anton Maximov, Franck Polleux

In the first paragraph of the Results section, the authors incorrectly refer to Fig S3D. The sentence should reference S5D, reading “Mitochondria are associated with about half of presynaptic sites in axons of mature pyramidal cortical neurons (S5D Fig) [21,29]”.
