# Supplementary figures and images for: LKB1 Regulates Mitochondria-Dependent Presynaptic Calcium Clearance and Neurotransmitter Release Properties at Excitatory Synapses along Cortical Axons
Source: PLoS Biol. 2016 Jul 18;14(7):e1002516. doi: 10.1371/journal.pbio.1002516 (PMC4948842; doi:10.1371/journal.pbio.1002516)

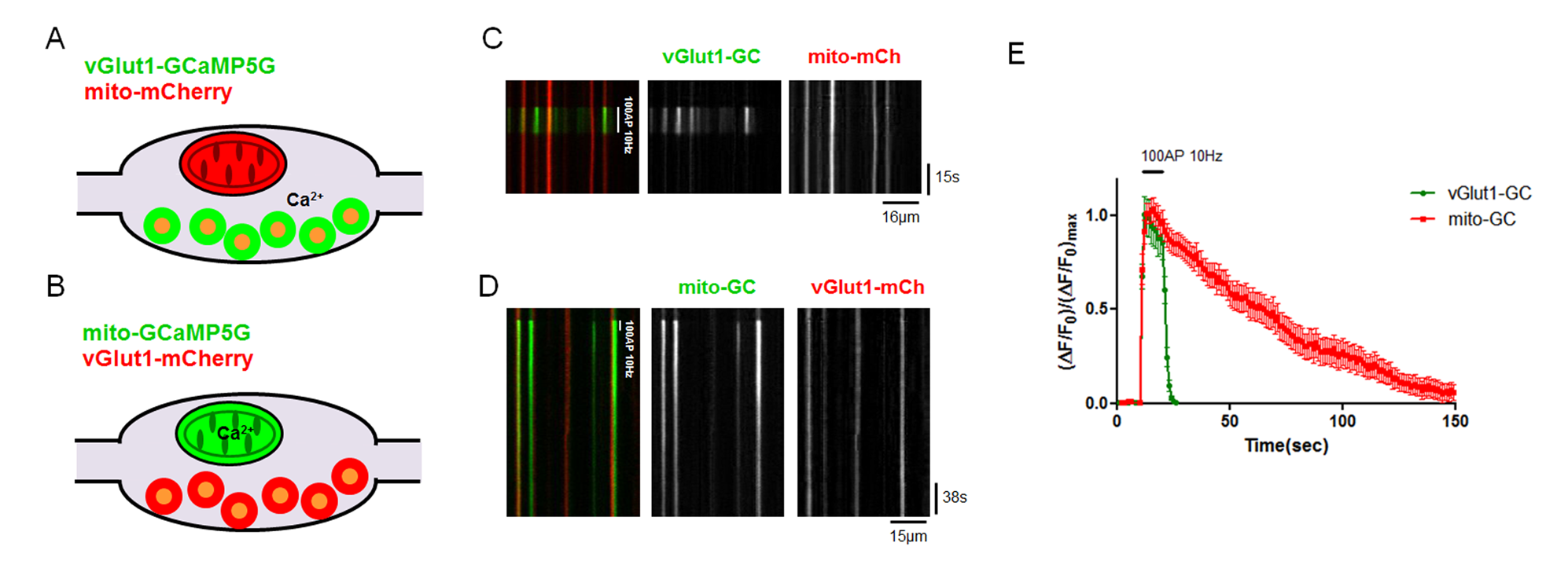

Supplement: S1 Fig — (A and C) Ca2+ dynamics at presynaptic sites occupied by mitochondria were monitored using expression of vGlut1-GCaMP5G and mito-mCherry in axons of cortical layer 2/3 neurons following ex utero electroporation at E15.5 followed by dissociation and culture for 15–17 DIV. (C) Representative kymograph of vGlut1-GCaMP5G (green) and mito-mCherry (red) before, during and after imposing 100 AP at 10 Hz (vertical white bar). (B and D) Calcium dynamics inside the matrix of mitochondria localized at a presynaptic bouton were measured using expression of mito-GCaMP5G and vGlut1-mCherry at the same condition. (B) Representative kymograph of mito-GCaMP5G (green) and vGlut1-mCherry (red) before, during and after imposing 100 AP at 10 Hz (white bar). (E) Intramitochondrial calcium influx shows significantly slower decay time than presynaptic Ca2+ clearance. Individual values are available in S1 Data. (TIF) [file pbio.1002516.s002.tif]

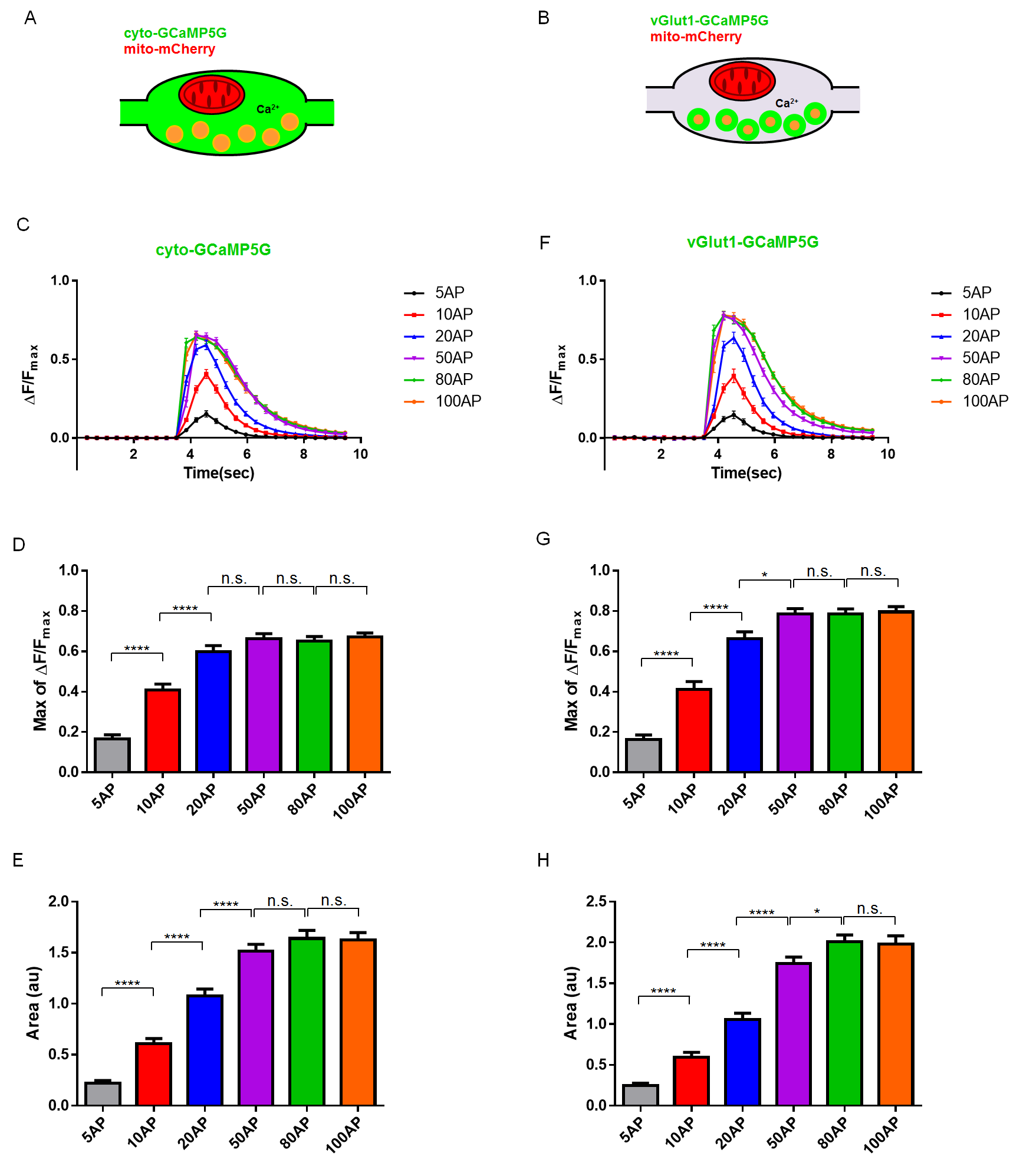

Supplement: S2 Fig — (A and B) vGlut1-GCaMP5G is spatially concentrated at presynaptic sites. (C-H) Cytosolic GCaMP5G and vGlut1-GCaMP5G signals were obtained by 1 s stimulation at different frequencies for given APs. Peak values of cytosolic GCaMP5G signals reached a maximum response at 20 AP, but vGlut1-GCaMP5G signals reached a maximum response at 50 AP, and total Ca2+ amount was reached maximum from 50 AP for cytosolic GCaMP5G, but from 80 AP for vGlut1-GCaMP5G. n = 14 for cyto-GCaMP5G, 12 for vGlut1-GCaMP5G. ** p < 0.01, *** p < 0.001. Mann-Whitney test. Individual values are available in S1 Data. (TIF) [file pbio.1002516.s003.tif]

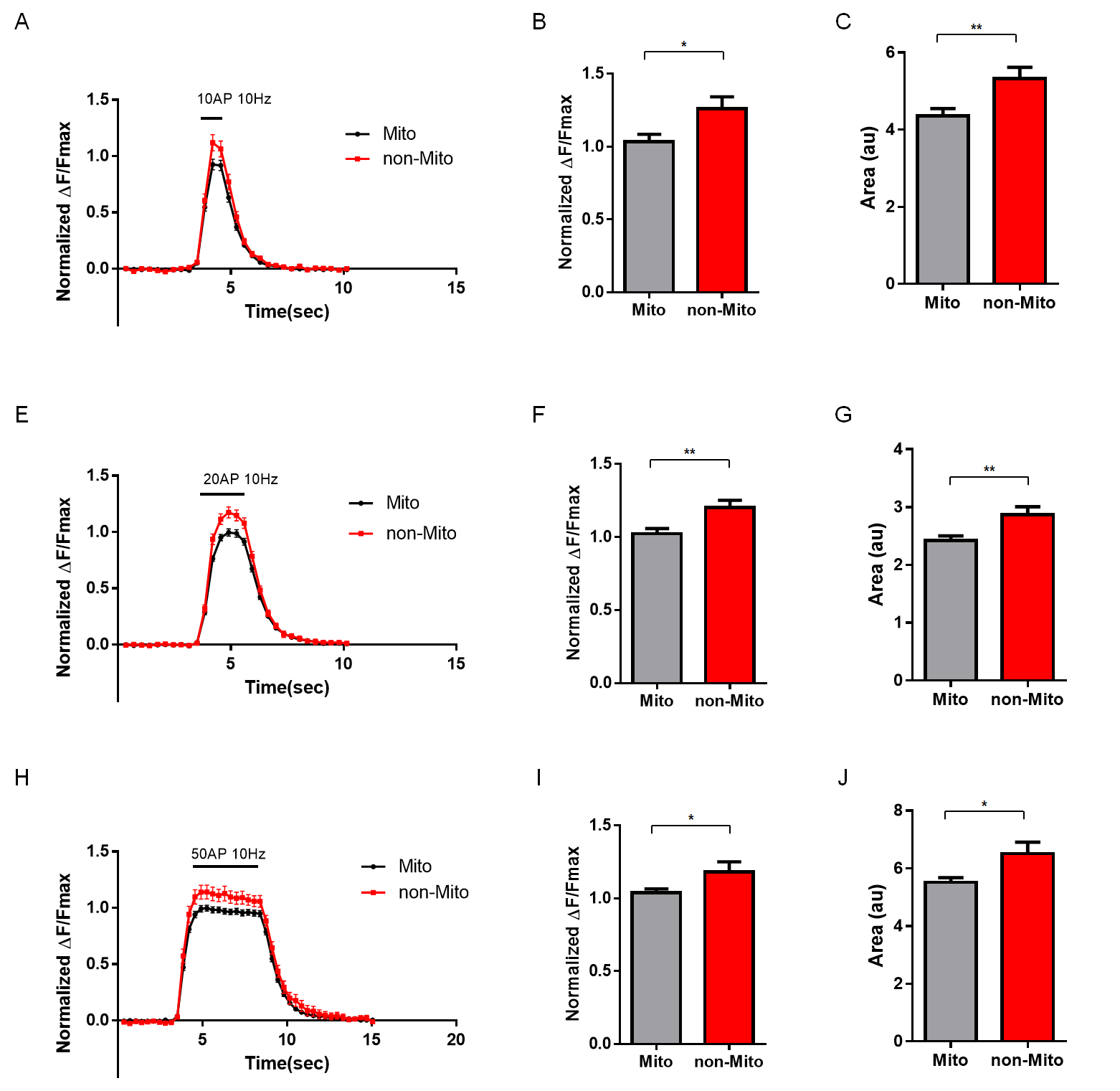

Supplement: S3 Fig — Presynaptic [Ca2+]c dynamics was measured at mitochondria-associated and mitochondria-free boutons using vGlut1-GCaMP5G and mito-mTagBFP in axons of cultured cortical neurons following ex utero electroporation at E15.5 and imaged at 15–17 DIV. Mitochondria-free boutons show significantly increased normalized peak values and total charge transfer (area under curve) during repetitive stimulation (10 AP, 20 AP, 50 AP, and 100 AP at 10 Hz). 10 AP: n = 62 for mito, and 43 for mito-free from 14 neurons. 20 AP: n = 62 for mito, and 40 for mito-free from 14 neurons. 50 AP: n = 47 for mito, and 30 for mito-free from 10 neurons. * p < 0.05 and ** p < 0.01, Mann-Whitney test. Individual values are available in S1 Data. (TIF) [file pbio.1002516.s004.tif]

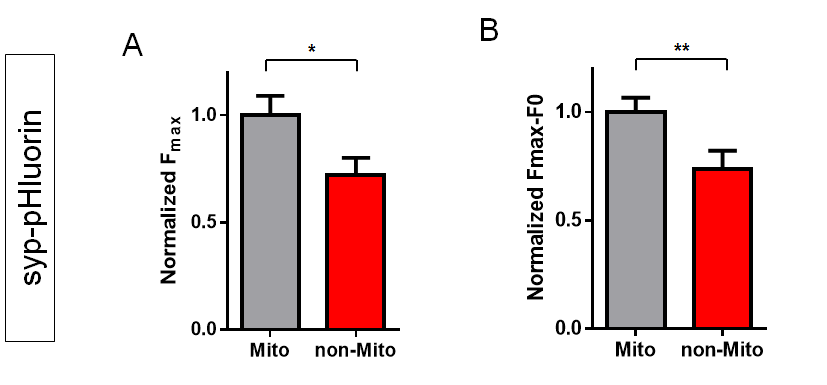

Supplement: S4 Fig — (A and B) Fmax values of syp-pHluorin was obtained by NH4Cl (50 mM) incubation, and both Fmax and Fmax-F0 values were significantly lower at mito-free than mitochondria-associated boutons. n = 26 for mitochondria-associated boutons and 15 for mitochondria-free boutons from 15 neurons in 20 AP condition. n = 31 for mitochondria-associated boutons and 17 for mitochondria-free boutons from 15 neurons in 100 AP condition. * p < 0.05, ** p < 0.01 Mann-Whitney test. Individual values are available in S1 Data. (TIF) [file pbio.1002516.s005.tif]

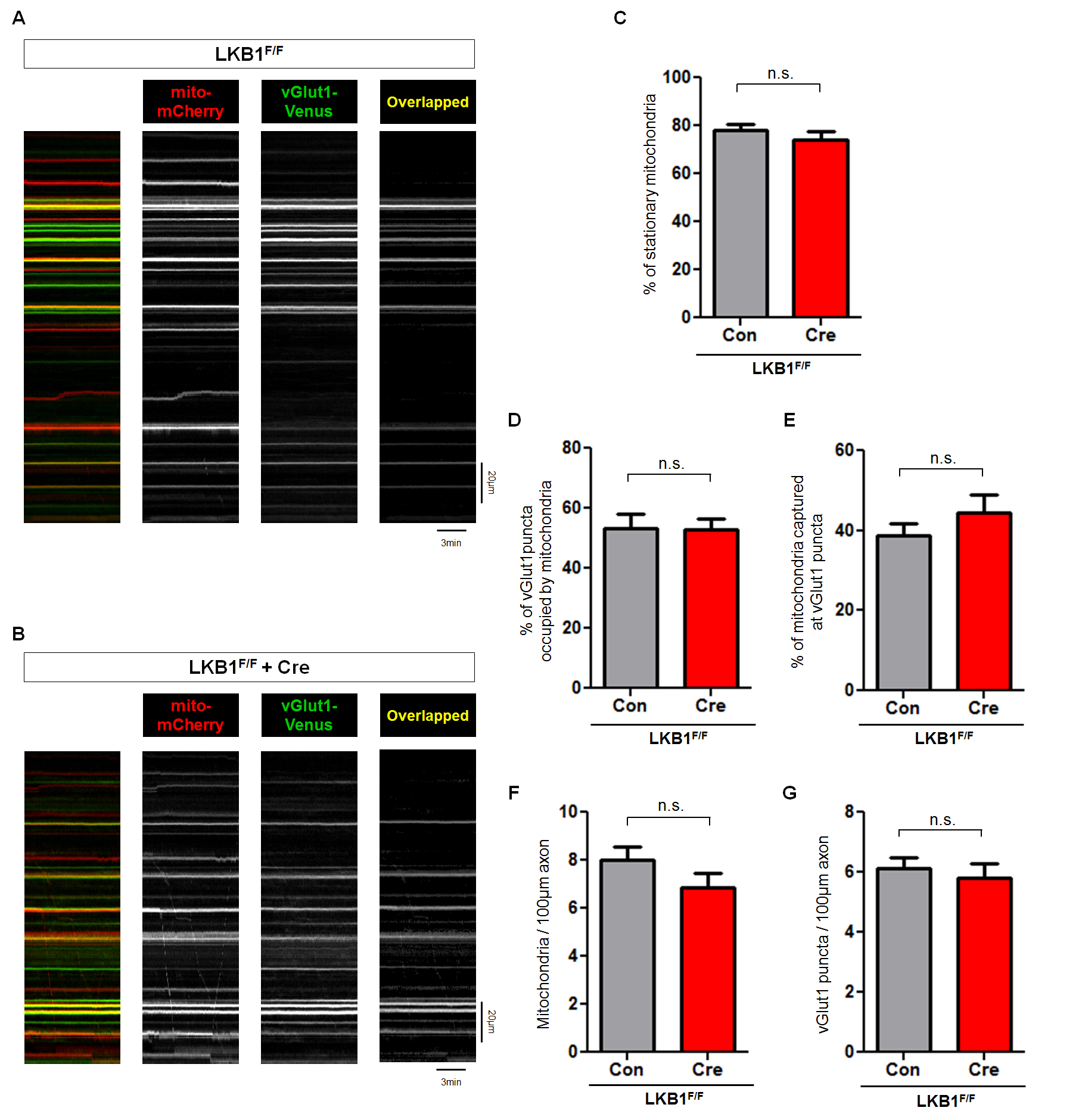

Supplement: S5 Fig — (A and B) Time-lapse microscopy of cortical neurons in culture reveals that mitochondria (labeled by Mito-mCherry (red)) occupy the same percentage of presynaptic boutons (vGlut1-Venus (green)) in WT and LKB1-mutant neurons at mature stage (E15.5+17DIV). Overlapping pixels maps shown in A and B were created in Fiji/ImageJ using the Colocalization Threshold plugin. (C) There was no significant difference in the percentage of stationary mitochondria between control and LKB1-deficient neurons. n = 24 for control, 25 for LKB1-deletion. (D and E) The percentage of vGlut1 puncta occupied by mitochondria as well as mitochondria captured at presynaptic sites was not altered in mature LKB1-deficient axons. n = 17 for control, 18 for LKB1-deletion. (F and G) Mitochondria and vGlut1 puncta density was also not altered in mature LKB1-deificient axons. n = 17 for control, 18 for LKB1-deletion. Mann-Whitney test. Individual values are available in S1 Data. (TIF) [file pbio.1002516.s006.tif]

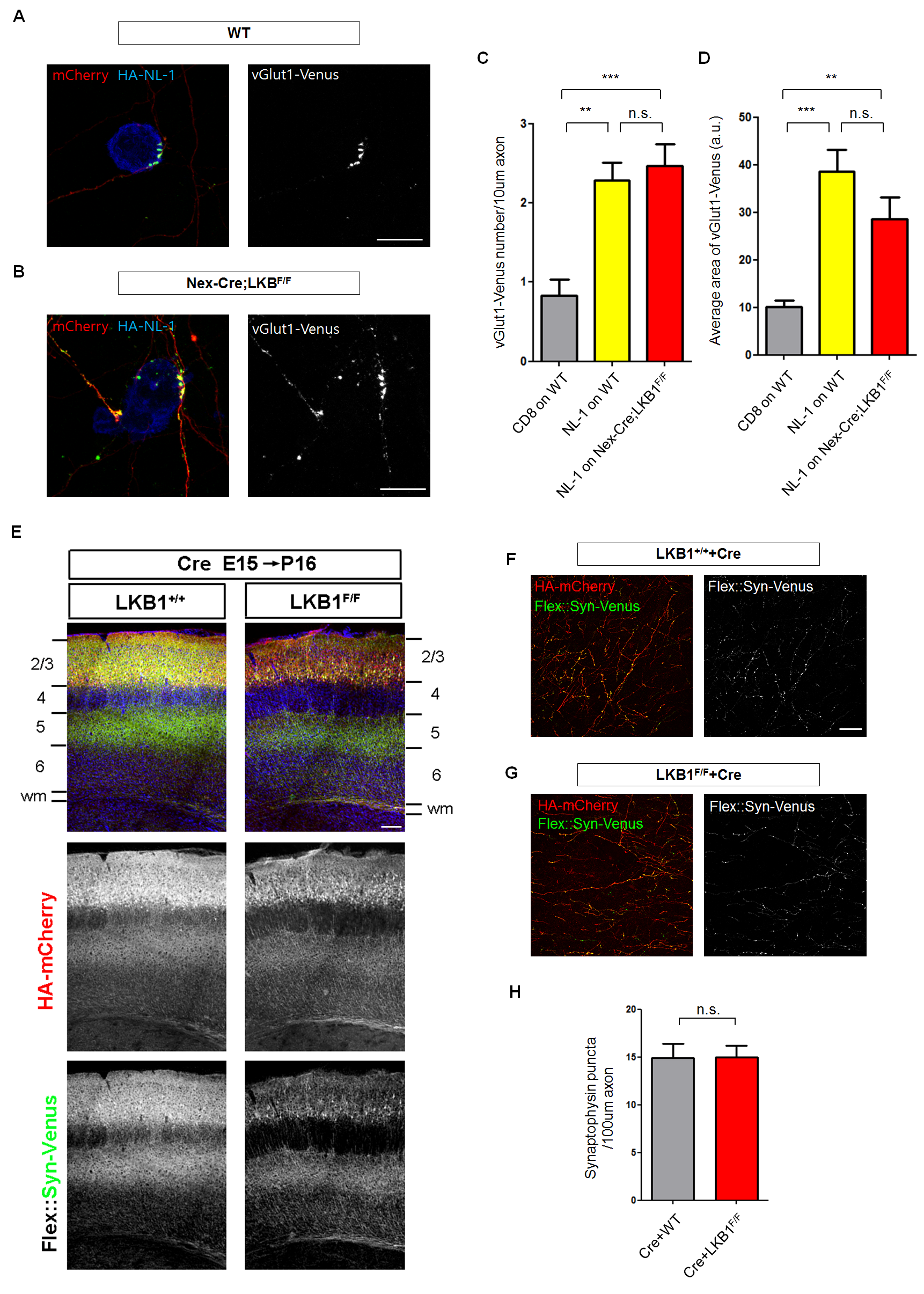

Supplement: S6 Fig — (A and B) HA-Neuroligin-1-expressing COS7 cells (blue) were co-cultured with WT and LKB1-null cortical neurons following ex utero electroporation at E15.5 and cultured for 9-12DIV. Single axons and their presynaptic boutons were visualized in cortical neurons by ex utero co-electroporation of mCherry and vGlut1-Venus by electroporation. Scale bar = 20 μm. (C) Quantification of the linear density of vGlut1-Venus-positive presynaptic boutons (#boutons/10 microns of axons growing over cells). Both WT and LKB1-null axons showed similar increased density of vGlut1-Venus puncta around neuroligin-1-expressing COS7 cells compared to cells transfected with CD8 (control). (D) Quantification of vGlut1-Venus-positive presynaptic bouton area formed over COS7 transfected with the indicated plasmids. Both WT and LKB1-null neurons had increased vGlut1-Venus area on the neuroligin-1-expressing COS7 cells. n = 16 for CD8 on WT, 16 for NL-1 on WT, and 16 for NL-1 on KO. ** p < 0.01, *** p < 0.001. Kruskal-Wallis test. (E) Plasmids encoding Cre recombinase, HA-mCherry and Flex::Synaptophysin-Venus were co-expressed by in utero cortical electroporation of mouse embryos at E15.5 (harvested at P16). Only Cre-expressing neurons could express synaptophysin-Venus signals following Cre-mediated inversion. LKB1-null neurons (right panels) displayed reduced density of synaptophysin-Venus staining compared to control (left panels) which is probably due to reduced terminal axon branching in ipsilateral layers 2/3 and 5, as previously reported [28]. Scale bar = 150 μm. (F and G) In high-magnification images of axon segments in hemisphere contralateral to the electroporation side, the linear density of synaptophysin-Venus presynaptic boutons was not altered in LKB1-deficient neurons compared to control. Scale bar = 30 μm. (H) Quantification of linear density of synaptophysin-Venus puncta (#boutons/microns). Total cumulative length of axon quantified = 2,831μm from 3 pups for control, 2,613 μm from [file pbio.1002516.s007.tif]

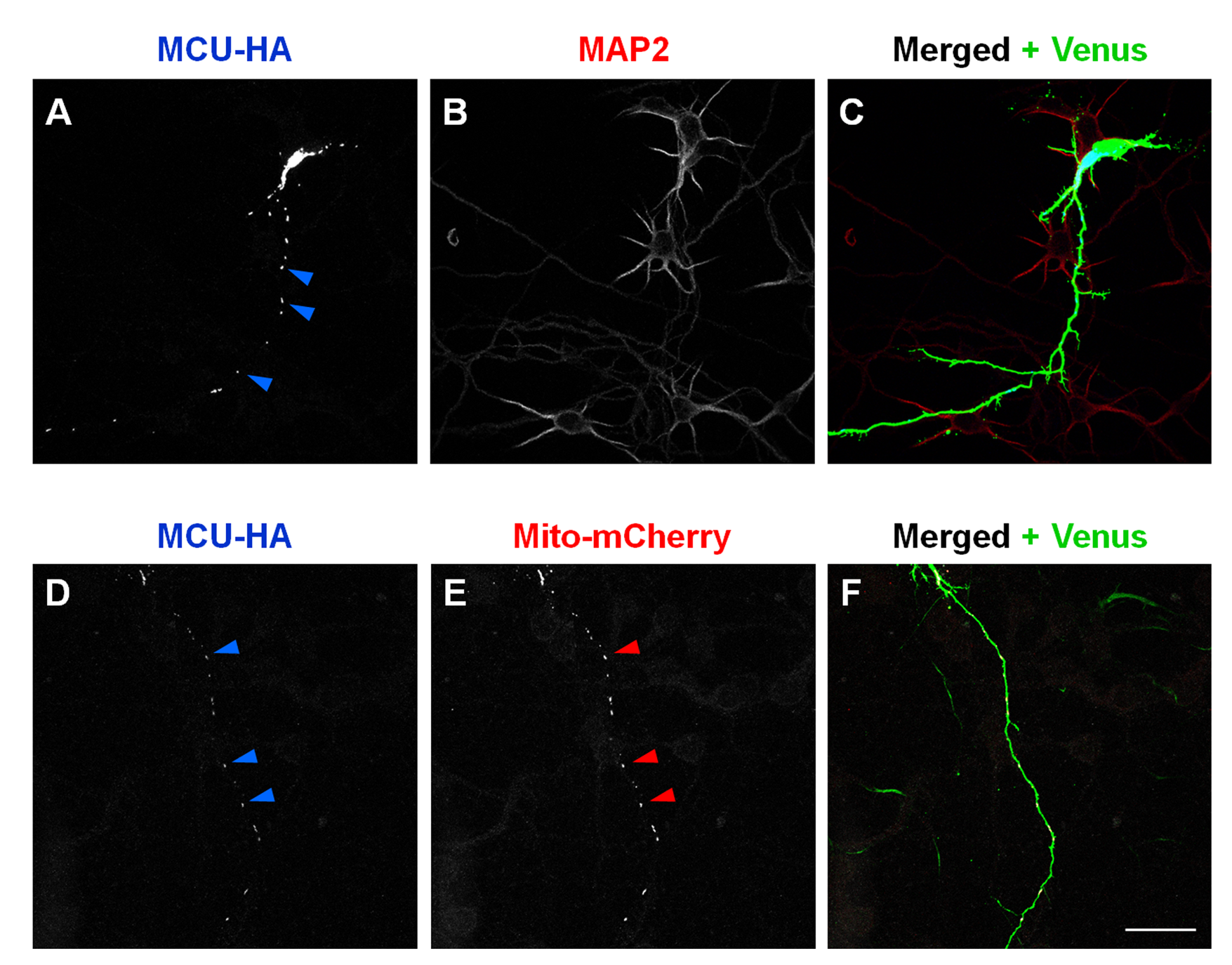

Supplement: S7 Fig — (A–C) Expression of HA-tagged MCU in cortical layer 2/3 neurons (ex utero electroporation, dissociated at E15.5 and cultured for 5 DIV) shows punctate distribution along the axon (MAP2-negative; arrowheads in A and B). (D–F) Co-expression of MCU-HA and Mito-mCherry in cortical layer 2/3 neurons (ex utero electroporation and dissociated at E15.5 + 5 DIV) reveals the co-localization of MCU with mitochondria throughout the neurons including the axon (arrowheads in D and E). Scale bar = 40 μm. (TIF) [file pbio.1002516.s008.tif]

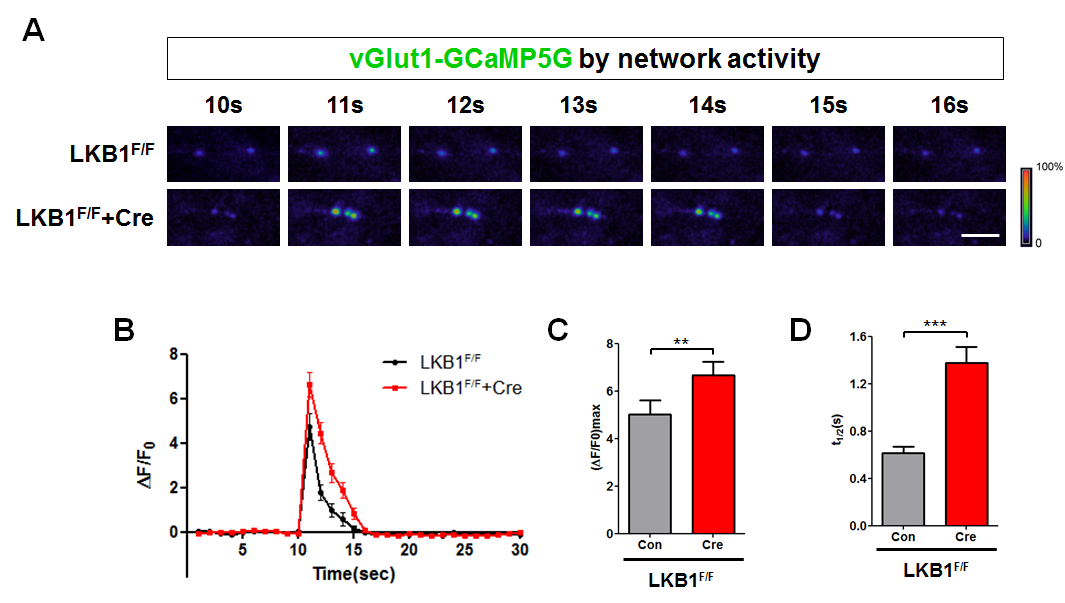

Supplement: S8 Fig — (A–D) Increased maximal presynaptic Ca2+ accumulation and delayed calcium clearance during spontaneous neurotransmitter release in layer 2/3 cortical pyramidal neurons. (A) Captured images from vGlut1-GCaMP5G timelapse series at presynaptic sites associated with mitochondria (mito-mCherry signal not shown). (B–D) Analysis of the temporal dynamics of vGlut1-GCaMP5G signals during spontaneous release (B) shows increased half-decay time (C) and increased maximum intensity (D) of [Ca2+]c in LKB1-deficient neurons (red) compared to control (gray). n = 35 for control from 12 neurons, 33 for LKB1–null from 11 neurons. *** p < 0.001 and ** p < 0.01, Mann-Whitney test. Scale bar = 10 μm. Individual values are available in S1 Data. (TIF) [file pbio.1002516.s009.tif]

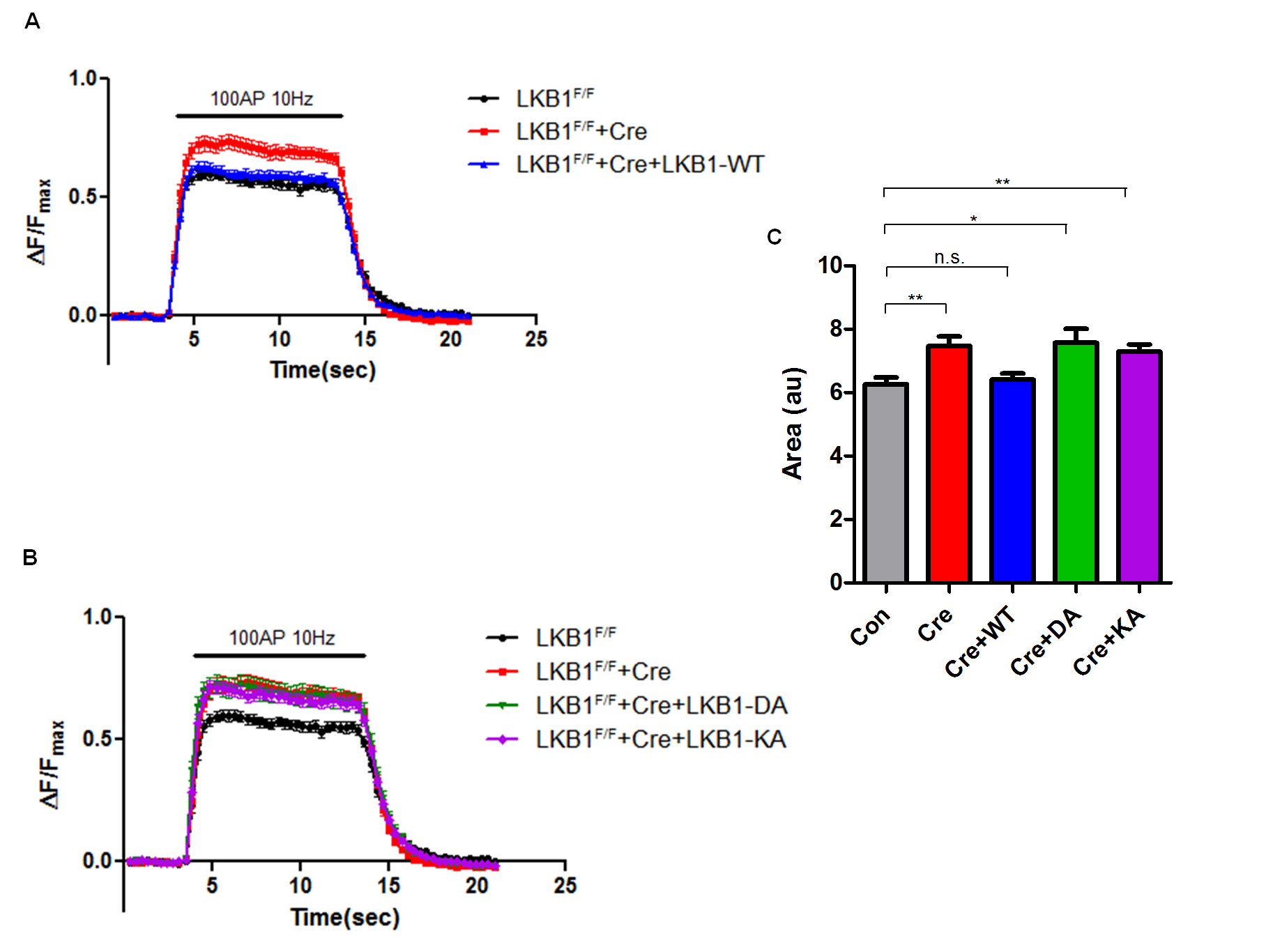

Supplement: S9 Fig — (A) Expression of wild-type LKB1 restores the elevated presynaptic Ca2+ observed in LKB1-deficient neurons back to control level. LKB1F/F cortical neurons were ex utero electroporated with control, Cre, and LKB1-WT (E15.5 + 16–17 DIV). (B) LKB1 kinase mutants, D194A and K78A, were not able to rescue calcium clearance defects on LKB1-null axons. (C) Total charge transfer (total area under curve) is analyzed from A and B. n = 32 for control, 36 for LKB1-deletion, 32 for LKB1-WT, 33 for LKB1-DA, 35 for LKB1-KA. * p < 0.05, ** p < 0.01, Mann-Whitney test. Individual values are available in S1 Data. (TIF) [file pbio.1002516.s010.tif]

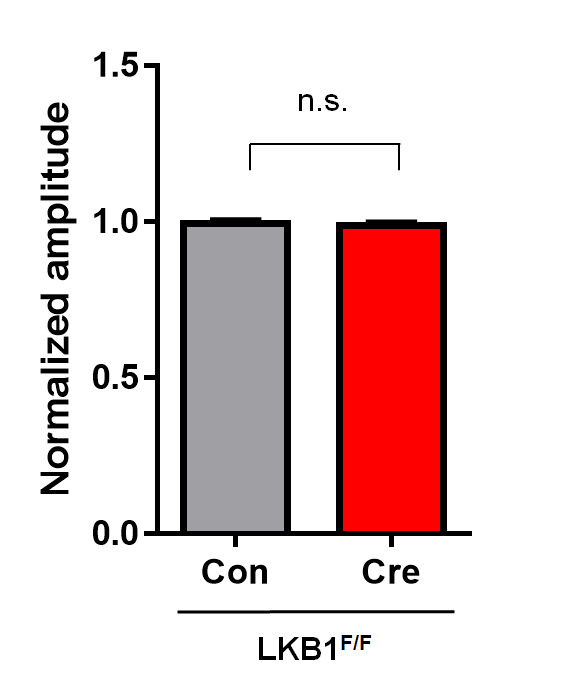

Supplement: S10 Fig — The amplitude of mEPSC measured by patch-clamp in LKB1-null neurons display non-significant difference compared to WT neurons. n = 4,012 events from 21 neurons for control and 4,401 events from 18 neurons for LKB1-null. Mann-Whitney test. Individual values are available in S1 Data. (TIF) [file pbio.1002516.s011.tif]

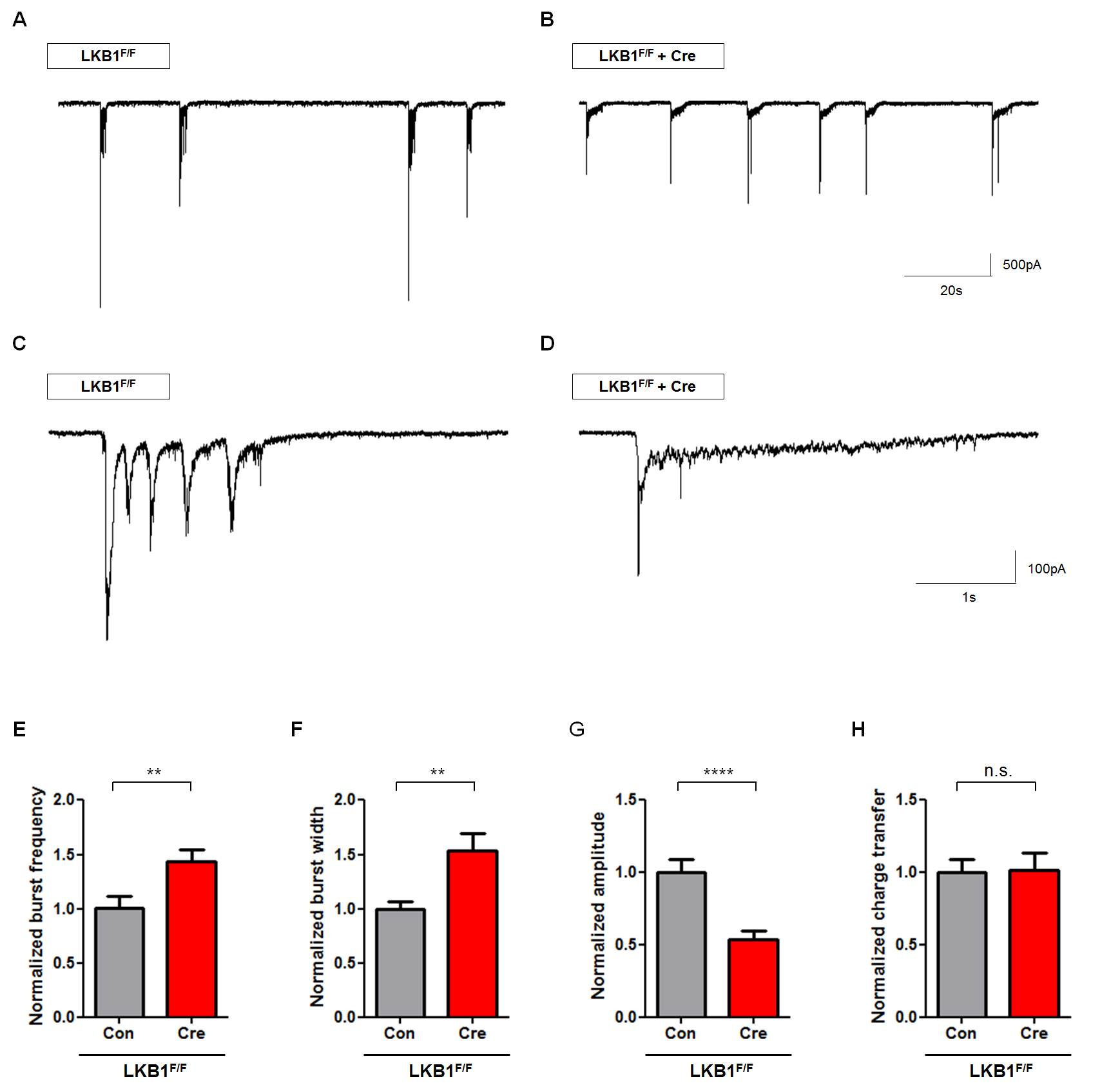

Supplement: S11 Fig — (A and B) LKB1-null neurons show more frequent burst firing but smaller amplitude. Dissociated cultures of LKB1F/F cortical neurons were infected with control or Cre-expressing lentivirus at 3–5 DIV and recorded at 15–18 DIV. (C and D) Analysis of single bursts reveals that LKB1-deficient neurons display longer burst width/duration, but similar charge transfer compared to control cortical neurons. (E–H) Quantification of burst frequency, width, amplitude and charge transfer. Data from each experiment were normalized by average of control values. n = 24 neurons for control, 29 for LKB1-deletion. ** p < 0.01, **** p < 0.0001, Mann-Whitney test. Individual values are available in S1 Data. (TIF) [file pbio.1002516.s012.tif]

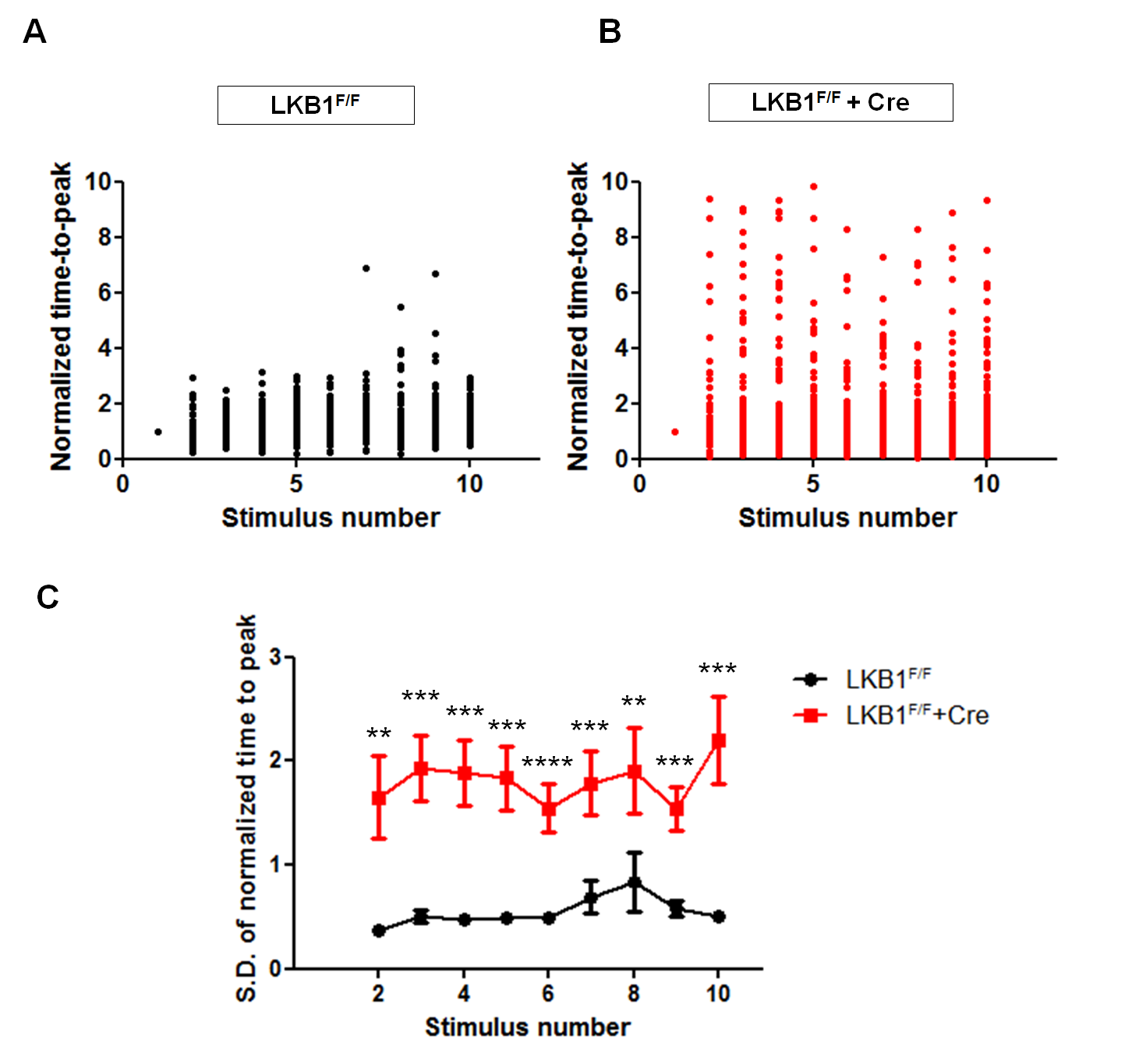

Supplement: S12 Fig — (A and B) Time-to-peak values from each EPSC peaks during 10 AP stimulation (10 Hz) were normalized by first response and plotted. (C) Standard deviation (SD) values of individual EPSCs are plotted from repetitive recordings (7–15) of each cortical neuron. Pooled data were analyzed with Mann-Whitney test. ** p < 0.01, *** p < 0.001, **** p < 0.0001. n = 28 for control, 27 for LKB1-deficient. Individual values are available in S1 Data. (TIF) [file pbio.1002516.s013.tif]

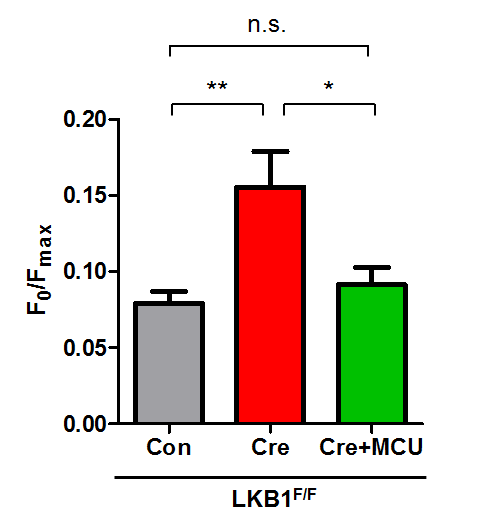

Supplement: S13 Fig — Basal presynaptic [Ca2+]c levels were monitored using vGlut1-GCaMP5G in LKB1-null and MCU-overexpressing LKB1-null axons at 15–17 DIV. Basal level of presynaptic [Ca2+]c is significantly elevated in LKB1-null axons compared to control cortical neurons, and MCU overexpression restores the increased basal Ca2+ to control levels. * p < 0.05, ** p < 0.01. Mann-Whitney test. n = 22 for control, 18 for LKB1-deficient, and 18 for LKB1-deficient + MCU. Individual values are available in S1 Data. (TIF) [file pbio.1002516.s014.tif]

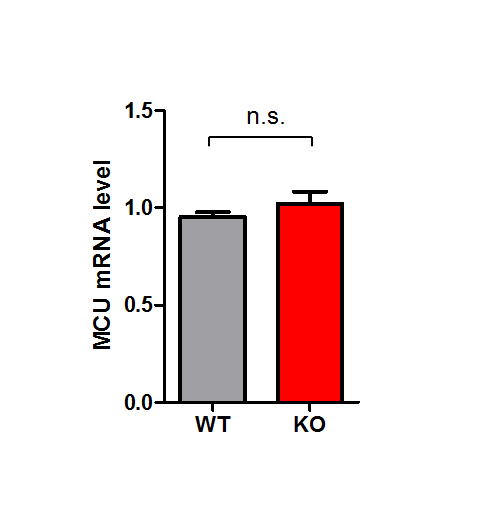

Supplement: S14 Fig — MCU mRNA was extracted and measured by RT-qPCR. Values were normalized by β-actin mRNA level. MCU mRNA level was not significantly changed in LKB1 knockout hippocampi (Nex-Cre;LKB1F/F, P19). Unpaired t test. Individual values are available in S1 Data. (TIF) [file pbio.1002516.s015.tif]
